# A Barth Syndrome Patient-Derived *D75H* Point Mutation in *TAFAZZIN* Drives Progressive Cardiomyopathy in Mice

**DOI:** 10.3390/ijms25158201

**Published:** 2024-07-27

**Authors:** Paige L. Snider, Elizabeth A. Sierra Potchanant, Zejin Sun, Donna M. Edwards, Ka-Kui Chan, Catalina Matias, Junya Awata, Aditya Sheth, P. Melanie Pride, R. Mark Payne, Michael Rubart, Jeffrey J. Brault, Michael T. Chin, Grzegorz Nalepa, Simon J. Conway

**Affiliations:** 1 Herman B. Wells Center for Pediatric Research, Indiana University School of Medicine, Indianapolis, IN 46033, USA; psnider@iu.edu (P.L.S.); esierra@iu.edu (E.A.S.P.); sunzejin1@yahoo.com (Z.S.); dmed@med.umich.edu (D.M.E.); kakuchan@iu.edu (K.-K.C.); adisheth@iu.edu (A.S.); ppride@iu.edu (P.M.P.); rpayne@iu.edu (R.M.P.); mrubartv@iu.edu (M.R.); gnalepa@iu.edu (G.N.); 2 Department of Anatomy, Cell Biology and Physiology, Indiana University School of Medicine, Indianapolis, IN 46202, USA; cmatias@iu.edu (C.M.); jebrault@iu.edu (J.J.B.); 3 Molecular Cardiology Research Institute, Tufts Medical Center, Boston, MA 02111, USA; j_awata@charter.net (J.A.); michael.t.chin@tuftsmedicine.org (M.T.C.)

**Keywords:** Barth syndrome, patient-tailored *Tafazzin* mutant allele, progressive cardiomyopathy, mitochondria, p53 pathway

## Abstract

Cardiomyopathy is the predominant defect in Barth syndrome (BTHS) and is caused by a mutation of the X-linked *Tafazzin (TAZ)* gene, which encodes an enzyme responsible for remodeling mitochondrial cardiolipin. Despite the known importance of mitochondrial dysfunction in BTHS, how specific *TAZ* mutations cause diverse BTHS heart phenotypes remains poorly understood. We generated a patient-tailored *CRISPR/Cas9* knock-in mouse allele (*Taz^PM^*) that phenocopies BTHS clinical traits. As *Taz^PM^* males express a stable mutant protein, we assessed cardiac metabolic dysfunction and mitochondrial changes and identified temporally altered cardioprotective signaling effectors. Specifically, juvenile *Taz^PM^* males exhibit mild left ventricular dilation in systole but have unaltered fatty acid/amino acid metabolism and normal adenosine triphosphate (ATP). This occurs in concert with a hyperactive p53 pathway, elevation of cardioprotective antioxidant pathways, and induced autophagy-mediated early senescence in juvenile *Taz^PM^* hearts. However, adult *Taz^PM^* males exhibit chronic heart failure with reduced growth and ejection fraction, cardiac fibrosis, reduced ATP, and suppressed fatty acid/amino acid metabolism. This biphasic changeover from a mild-to-severe heart phenotype coincides with p53 suppression, downregulation of cardioprotective antioxidant pathways, and the onset of terminal senescence in adult *Taz^PM^* hearts. Herein, we report a BTHS genotype/phenotype correlation and reveal that absent Taz acyltransferase function is sufficient to drive progressive cardiomyopathy.

## 1. Introduction

The heart is the most metabolically active organ, with only enough intracellular adenosine triphosphate (ATP) to support a few seconds of contractions [1]. To overcome this limitation, cardiomyocyte (CM) mitochondria are the most populous and generate >95% of the ATP required to maintain cardiac output [1,2,3]. Additionally, cardiac mitochondria play important roles in regulating redox status, Ca^2+^ homeostasis, lipid synthesis, intracellular survival, and oxidative stress repair [1,2,3]. Consequently, cardiomyopathy is common among mitochondriopathies, with the most frequent manifestation being dilated cardiomyopathy (DCM) [3,4]. Barth syndrome (BTHS; OMIM #302060) is a rare, infantile-onset, X-linked mitochondriopathy that exhibits a variable presentation of failure to thrive, short stature, skeletal myopathy, neutropenia, and heart anomalies due to mitochondrial dysfunction secondary to inherited *TAFAZZIN* (*TAZ*; OMIM #300394) mutations [5,6,7]. Cardiomyopathy is the most common BTHS clinical feature and the primary driver of disease outcomes that include DCM, left ventricular non-compaction (LVNC), heart failure (HF), and/or arrhythmia [5,6,7,8]. As with most mitochondriopathies, there is no cure for BTHS, and most male BTHS patients succumb to heart failure (HF), arrhythmias, sepsis, and premature death [7,8,9].

*TAZ* is present on the X-chromosome [6] and is an acyl-specific H(X)_4_D transferase whose primary enzymatic function is the remodeling of immature monolysocardiolipin (MLCL) to mature cardiolipin (CL), an essential component of the mitochondrial membrane [4,9,10]. Accordingly, *TAZ* loss-of-function mutations result in decreased amounts of CL, a buildup of precursor MLCL, and diminished mitochondrial oxidative phosphorylation (OxPhos) [8,9,10]. CL is a phospholipid unique to mitochondria [11], and MLCL is usually not detectable in normal healthy tissues [12]. CL is enriched in CMs and critical for mitochondrial structural integrity, permeability, and cristae morphogenesis, as well as directly interacting with the electron transport chain (ETC) complexes required to synthesize ATP [2,11]. In addition to its acyltransferase motif, *TAZ* also encodes mitochondrial localization and membrane anchoring domains, as well as a unique hydrophilic domain that may serve as an exposed loop interacting with additional unidentified proteins [9,13]. TAZ itself may sense mitochondrial membrane curvature and play a direct role in cristae reorganization and stability [11,12,13]. Despite the known importance of mitochondrial function and CL remodeling in BTHS as well as in cardiovascular health within the general population [13], how *TAZ* mutations cause the variety of divergent and fluctuating heart phenotypes seen in BTHS patients is poorly understood [4,8,14] This stems from an incomplete understanding of BTHS-affected downstream signaling, inconsistent genotype-phenotype correlations, and a lack of patient-tailored mouse models to interrogate mutation-specific effects [9,15].

Clinically, >200 unique single nucleotide missense, nonsense, or frameshift mutations within *TAZ*, rather than large deletions or chromosomal changes, have been identified as causative of BTHS [https://www.barthsyndrome.org accessed 4 March 2024]. Moreover, in vitro characterization of *TAZ* variants has demonstrated that these alleles frequently express mutant proteins with variable phenotypes ranging from benign to potent loss-of-function phenotypes [16,17,18,19]. However, in vivo BTHS cardiopathologic studies have been greatly hindered by the absence of mouse models that express mutant *Taz* protein, as existing *Taz* mouse models either totally lack Taz protein [8,18,19] or express doxycycline-induced diminished levels of wild-type Taz protein [20].

Herein, we report our identification of a novel de novo *TAZ* variant, causing a *D75H* substitution within the H(X)_4_D acyltransferase motif, in a BTHS patient presenting with cardiomyopathy, muscle wasting, short stature, and neutropenia. Taking advantage of this residue’s conservation [6], we used *CRISPR/Cas9* genome editing to knock-in this patient’s mutation into the murine *Taz* locus (*Taz^PM^*). Significantly, surviving male *Taz^PM^* mice express a stable mutant Taz^D75H^ protein and faithfully recapitulate the patient’s disease spectrum, revealing that loss of Taz transacylase activity is sufficient to phenocopy the progressive cardiomyopathy characteristic of BTHS. Further, we demonstrate that the changeover from a mild-to-severe heart phenotype coincides with the suppression of distinct juvenile cardioprotective effectors prior to subsequent pathological HF and premature death in adult male *Taz^PM^* mice. Thus, this novel patient-tailored *Taz^PM^* mouse model provides a clinically relevant tool for in vivo interrogation of the mechanisms underlying BTHS pathogenesis as well as a preclinical platform for the development of precision treatment strategies for BTHS.

## 2. Results

### 2.1. Point Mutant Taz^PM^ Mice Recapitulate Barth Syndrome Clinical Characteristics

**Figure 1:**
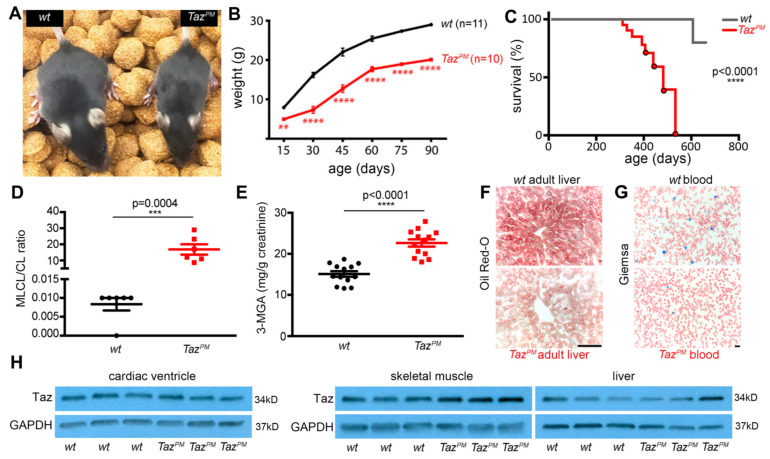
Phenotyping *Taz^PM^*♂ point mutant knock-in mice. (**A**) *Taz^PM^*♂ next to larger *wt*♂ (left) littermate at 1 month of age. (**B**) Between 1 and 3 three months of age, *Taz^PM^*♂ on average are 4–6 g < *wt*♂ littermates (*n* = 10 *Taz^PM^*♂ and 11 *wt*; *p* < 0.0001). (**C**) Kaplan–Meier survival curves of age-matched *wt*♂ (*n* = 82) and *Taz^PM^*♂ (*n* = 29) reveal curtailed *Taz^PM^*♂ lifespans (*p* < 0.0001). (**D**) Mass spectrometry quantification of MLCL:CL ratio in blood from 4-month *Taz^PM^*♂ vs. *wt*♂ (*n* = 6/genotype) confirms deficient CL biosynthesis. (**E**) Relative 3-methylglutaconic acid levels/gram of creatinine in *Taz^PM^*♂ and *wt*♂ urine (*n* = 13/genotype). (**F**) Oil red-O stain reveals diminished microvesicular lipid deposition in *Taz^PM^*♂ (lower panel) but not in *wt*♂ (upper panel) littermate adult livers (n = 17 litters). (**G**) Giemsa stain of juvenile *Taz^PM^*♂ peripheral blood revealed a <50% reduction in neutrophils compared to *wt*♂. (**H**) Western analysis of steady-state Taz levels in surviving adult *Taz^PM^*♂ (*n* = 6) and *wt*♂ (*n* = 7) littermate ventricles (atria and valvular tissues removed), gastrocnemius skeletal muscles and livers, normalized to housekeeping GAPDH levels. Quantification and statistical analysis are shown in Supplemental Figure S1. Statistical significance set at ** *p* < 0.01, *** *p* < 0.005 and **** *p* < 0.001. Scale bars: (**F**) = 500 μm, (**G**) = 20 μm.

We identified a novel *TAZ* variant at c.223, causing a *D75H* substitution in a BTHS patient presenting with recurrent fevers, neutropenia, gross motor delay, and hypotonia in a 1.5-year-old boy. This BTHS patient was diagnosed with severe non-compaction cardiomyopathy at birth. Subsequent analysis at 6 years (study ID 6) revealed an elevated MLCL/CL ratio, reduced height/weight Z-score, and significantly lower cardiac fractional shortening [21]. This phenotypically, biochemically, and molecularly confirmed that the BTHS *Asp75His* variant is classified pathogenic by PolyPhen-2 [22] as the affected aspartate lies in the TAZ enzyme’s critical H(X)_4_D acyltransferase active site (Figure S1A). We used *CRISPR/Cas9* genome editing to introduce this patient’s mutation into the murine *Taz* locus, thereby generating a unique patient-tailored *Taz^PM^* (point mutation) allele (Figure S1B). Genotyping (Figure S1C) revealed *Taz^PM^* males (♂) are born at <expected Mendelian frequency (38/120 genotyped ♂; *p* < 0.0001 in Fisher’s exact test), but *Taz^PM^/X* females (♀) are born at expected ratios and are grossly indistinguishable from *wt*♀ at weaning (Table 1). Consistent with stillbirths and prenatal loss associated with BTHS [7,8,20], we found that only ~50% of *Taz^PM^*♂ survive to adulthood. Growth curve analysis revealed surviving *Taz^PM^*♂ exhibit sustained growth restriction and consistently weigh ~50% less than *wt*♂ littermates (Figure 1A,B). Like BTHS patients [6,7], surviving adult *Taz^PM^*♂ remain runted throughout life (*n* = 42/42). Moreover, Kaplan–Meier analysis demonstrated that *Taz^PM^*♂ exhibit a decreased lifespan following a period of dyspnea and decreased activity; 100% of *Taz^PM^*♂ died by ~550 days, at which point no *wt*♂ had died (Figure 1C). Significantly, mass spectrometry revealed an increased ratio of intermediary monolysocardiolipin (MLCL) relative to mature cardiolipin (C18:2_4_) ratio in *Taz^PM^*♂ compared to *wt*♂ (15 and 0.008 MLCL:CL ratio, respectively; *p* < 0.05), reflecting an accumulation of MLCL and a CL decrease (Figure 1D). 

Clinically, impaired CL biosynthesis is a highly sensitive and universal diagnostic for BTHS [2,10,11,12,13]. Thus, we generated a human cDNA carrying the *D75H* substitution mutation to test its ability to remodel CL directly in *Taz*-deficient MEFs. As expected, the stably expressed TAZ^D75H^ mutant protein is unable to rescue the increased MLCL/CL ratio in *Taz* null cells, verifying that both human and mouse D75H mutant proteins lack acyltransferase enzyme activity (Figure S1D,E). Combined with the elevated in vivo MLCL:CL ratio, these data suggest Taz^D75H^ enzyme activity is attenuated, but future studies are required to determine if there are any changes in the synthesis and/or degradation of these lipids or whether de novo biosynthesis of CL is impaired. Adult *Taz^PM^*♂ mice also exhibited a ~50% increase in 3-methylglutaric acid (3-MGA) in their urine as compared with *wt*♂ (*p* < 0.05; Figure 1E), which is another clinical indicator of BTHS [10]. Additionally, all adult *Taz^PM^*♂ livers (*n* = 13/13) had decreased staining for lipid content compared to *wt*♂ littermates (Figure 1F), indicative of liver dysfunction [23]. Complete blood counts revealed a <50% reduction in juvenile 4-week-old *Taz^PM^*♂ neutrophils (Figure 1G) and reduced neutrophil Ly-6GC expression, indicating mild neutropenia (*p* = 0.04 by *t*-test; *n* = 11/genotype), whereas 2/11 had absolute neutrophil counts as low as 200/mm^3^, reflecting full neutropenia. However, in 4- and 10-month-old adults, *Taz^PM^*♂ complete blood cell counts and Ly-6GC expression were comparable to *wt*♂, reflecting the inherent variability often observed in BTHS [6,7]. Significantly, surviving *Taz^PM^*♂ express a mutant Taz^D75H^ protein at equivalent levels to *wt*♂ in multiple adult organs, including the heart, skeletal muscle, and liver (Figure 1H and Figure S1F), phenocopying what is observed in most BTHS patients [13,14,15]. Similarly, *Taz* mRNA levels are equivalent in *Taz^PM^*♂ and *wt*♂ littermate hearts (Figure S1G), confirming unchanged *Taz^D75H^* transcription as well as translation. Thus, we generated the first BTHS patient-specific mouse model that expresses a stable mutant Taz^D75H^ protein. Collectively, these phenotypic and biochemical characterization data support the designation of the *Taz^PM^* point mutant allele as a patient-specific phenotypical syndromic mouse model that recapitulates the major clinical features observed in BTHS male patients.

### 2.2. Adult Cardiac Phenotype in Surviving Taz^PM^ Male Mice

**Figure 2:**
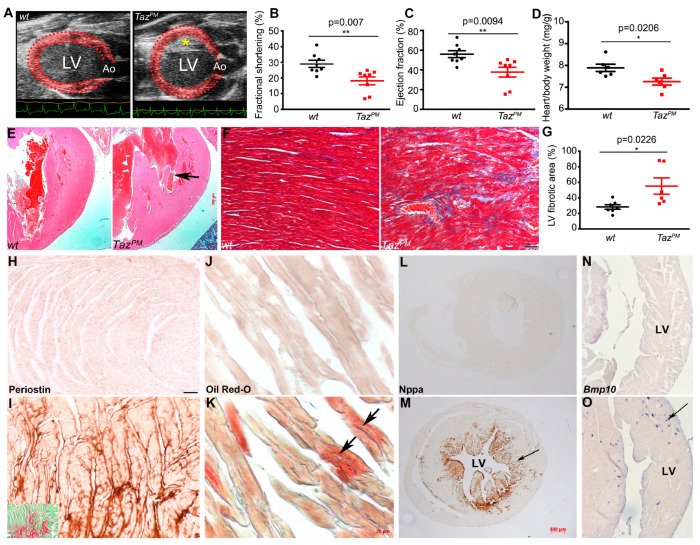
Adult *Taz^PM^*♂ cardiac phenotype. (**A**) Transthoracic echocardiographic measurements (*n* = 8/genotype) reveal a dilated “pumpkin-shaped” LV in 10-month *Taz^PM^*♂ as compared to *wt*♂ littermates. Prominent *Taz^PM^*♂ LV trabeculations indicated via *. (**B**,**C**) Quantification demonstrates both decreased fractional shortening ((**B**), *p* = 0.007) and ejection fraction ((**C**), *p* = 0.009) in *Taz^PM^*♂ (red) compared to *wt*♂ (black) littermates (*n* = 8/genotype). (**D**) 10-month heart/body weight measurements show a reduction from ~8 mg/g in *wt*♂ to ~7.1 mg/g in *Taz^PM^*♂ (*n* = 6/genotype, *p* = 0.02). (**E**) Histology confirmed all 10-month *Taz^PM^*♂ hearts (*n* = 5/5) exhibit significant ventricular chamber dilatation with prominent LV trabeculations with deep intertrabecular recesses (arrow) compared to *wt*♂ littermates. (**F**) Masson’s trichrome (*n* = 6/genotype) revealed increased fibrosis (blue) in *Taz^PM^*♂ but not *wt*♂ LV. (**G**) Morphometric measurement of fibrosis (*n* = 6/genotype) as % of the entire LV myocardium. (**H**,**I**) Cardiac fibrosis was confirmed via immuno-histochemical detection of ectopic deposition of profibrotic Periostin (brown) in 10-month *Taz^PM^*♂ ((**H**), *n* = 6/6) compared to *wt*♂ ((**I**)) littermate hearts. Sirus Red/methyl green inset (**I**), wherein red indicates fibrosis overlapping Periostin expression. (**J**,**K**) Oil Red-O (*n* = 6/genotype) reveals significant steatosis (red) in only adult *Taz^PM^*♂ CMs ((**K**), arrows). (**L**,**M**) Immunohistochemistry revealed Nppa protein (brown) is ectopically upregulated within mainly the adult *Taz^PM^*♂ trabecular LV zone (**M**, indicated by arrow) but absent in *wt♂* littermates (**L**). (**N**,**O**) Non-radioactive in situ hybridization revealed ectopic *Bmp10* mRNA is induced in adult *Taz^PM^*♂ ventricle cardiomyocytes ((**O**), arrow). Statistical significance set at * *p* < 0.05 and ** *p* < 0.01. Abbreviations: LV, left ventricle. Scale bars: (**E**) = 200 μm, (**F**,**H**,**I**) = 100 μm, (**J**,**K**) = 20 μm, (**L**,**M**) = 500 μm.

Because striated muscle mitochondria are uniquely enriched for the remodeled CL species (C18:2)_4_, mitochondriaare severely impacted by TAZ deficiency, and BTHS is predominately characterized by cardiomyopathy and hypotonia [7,8,9,10,11,12], we focused on examining the effects of acyltransferase deficiency in surviving *Taz^PM^*♂ hearts. Although ~50% of *Taz^PM^*♂ do not survive the neonatal phase, echocardiography of surviving adult *Taz^PM^*♂ revealed a dilated “pumpkin” shape (Figure 2A), which is frequently found in left ventricular (LV) non-compaction hearts (LVNC) due to a lack of a definitive ventricular apex [4]. Despite the altered geometry of the mutant LV chambers, both LV fractional shortening (FS) and ejection fraction (EF) were significantly reduced in *Taz^PM^*♂ as compared to *wt*♂ mice (Figure 2B,C; Table 2), indicating LV systolic dysfunction. Moreover, LV internal diameters during both systole and diastole were significantly smaller in *Taz^PM^*♂ versus *wt*♂ (Supplemental Figure S2A,B and Table 2) but heart rates were unchanged (*wt*♂ 518 ±11 vs. *Taz^PM^*♂ 487 ±32 beat/min; *n* = 8/genotype). Both *Taz^PM^*♂ LV mass (Figure S2C) and overall heart weight normalized to body weight were also significantly reduced (Figure 2D). Histology confirmed adult *Taz^PM^*♂ hearts exhibit prominent LV trabeculations with deep intertrabecular recesses (Figure 2E) consistent with LVNC. Adult *Taz^PM^*♂ hearts also exhibit cardiac fibrosis (Figure 2F,G) and increased profibrotic matricellular Periostin [24] deposition interstitially in *Taz^PM^*♂ ventricles (Figure 2I) with no evidence of myocyte hypertrophy (Figure S2E). Oil Red-O staining also revealed abnormal lipid deposition (steatosis) in *Taz^PM^*♂ CMs, that was not seen in *wt*♂ (Figure 2J,K).

At the molecular level, we found canonical cardiac remodeling *Nppa* mRNA and protein (stress-induced atrial natriuretic factor) are ectopically induced in adult *Taz^PM^*♂ hearts, predominantly in the LV trabecular-derived zone (Figure 2M and Figure S2G), indicating chamber-restricted stress induction. As Nppa modifies lipid and carbohydrate metabolism, its upregulation in mutants suggests stimulation of anaerobic glycolysis. Consistent with myocardial stress, the trabecular CM-restricted LVNC-associated TGFβ superfamily member *Bmp10* peptide [4] is also ectopically upregulated (Figure 2O). As apoptosis can initiate a fibrotic response [25] and lipid accumulation can drive lipoapoptosis [26], we assessed apoptosis at various ages. Surprisingly, we did not detect any increase in TUNEL positivity in juvenile or adult *Taz^PM^*♂ hearts (Figure S2I). Collectively, these data indicate that surviving adult *Taz^PM^*♂ hearts exhibit an LVNC phenotype that undergoes pathological cardiac fibrosis and LV systolic dysfunction, accompanied by poor somatic growth and premature lethality. Indeed, LVNC, alone or in combination with other heart anomalies, occurs in ~50% of BTHS patients [15], and BTHS hearts can exhibit cardiac fibrosis [8].

### 2.3. Progressive Cardiomyopathy in Taz^PM^ Males

**Figure 3:**
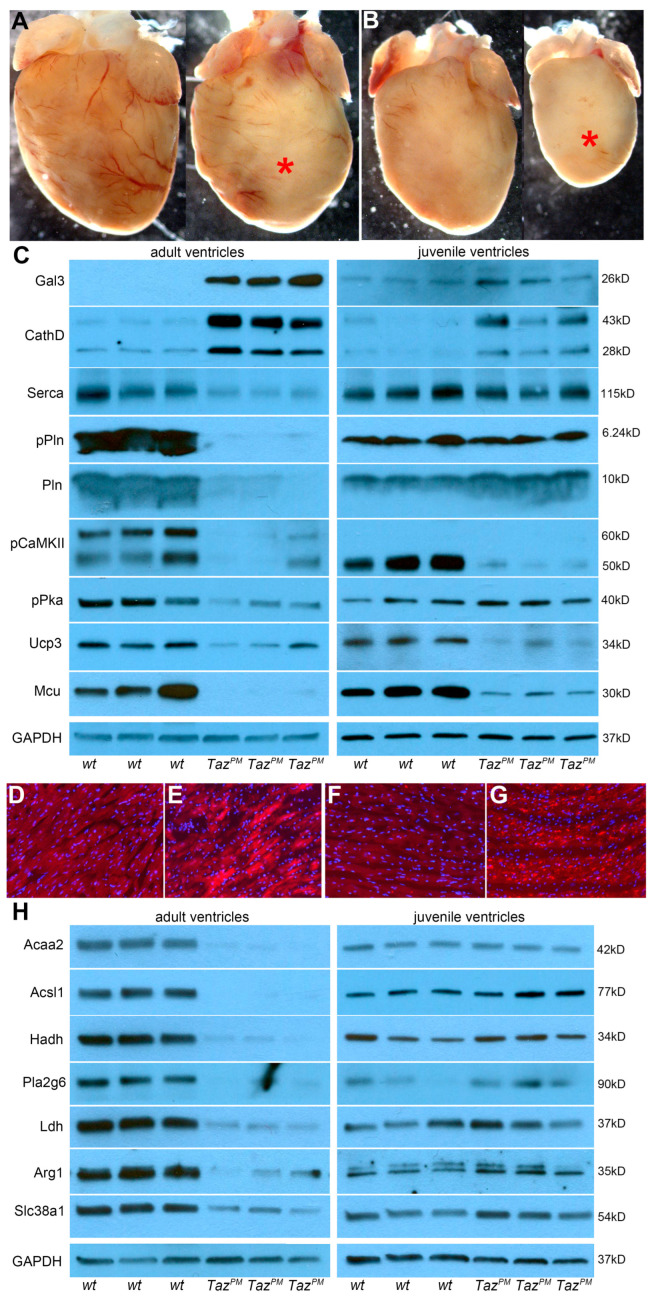
Progressive metabolism dysfunction and heart failure in *Taz^PM^* males. (**A**,**B**) Representative images of whole adult (**A**) and juvenile (**B**) littermate *wt*♂ and *Taz^PM^*♂ (red *) hearts. (**C**) Western analysis using antibodies against key profibrotic and cardiac output prognostic biomarkers in adult (6–8 months) and juvenile (4 weeks) *wt*♂ and *Taz^PM^*♂ ventricles. (**D**–**G**) Fluorescent immunohistochemistry revealed Gal3 and CathD (red) are both upregulated in *Taz^PM^*♂ cardiomyocytes (**E**,**G**) but are absent in *wt*♂ adult littermates (**D**,**F**). Nuclei counterstained with DAPI (blue). (**D**–**G**) were magnified ×200. (**H**) Western analysis using antibodies against fatty acid and amino acid metabolism effectors. GAPDH was used as a loading control and triplicate age-matched adult and juvenile *wt*♂ (*n* = 7) and *Taz^PM^*♂ (*n* = 6) littermate ventricles were examined. Quantification and statistical analysis are shown in Supplemental Figure S4.

Surviving adult *Taz^PM^*♂ grow poorly and start to die at ~10 months of age (Figure 1C), thus we examined both surviving juvenile (4–8 weeks) and adult (6–8 months) *Taz^PM^*♂ hearts prior to and during overt cardiomyopathy. Gross examination reveals juvenile *Taz^PM^*♂ hearts are ~31.6% undersized (Figure 3B), but adult *Taz^PM^*♂ hearts are only ~11.25% smaller than *wt*♂ littermates (Figure 3A) when normalized against body weights. Echocardiography shows significantly diminished EF and FS in juvenile *Taz^PM^*♂ versus *wt*♂ mice (Figure S3A; Table 2). Notably, the magnitude of EF was significantly larger in juvenile *Taz^PM^*♂ mice than seen in their adult counterparts despite similar heart rates (Table 2), suggesting regression of LV dilation with age, which is consistent with the clinical course seen in BTHS patients. Further, hearts from juvenile *Taz^PM^*♂ mice exhibit LV chamber dilation in end-systole but not end-diastole (Figure S3B,D), consistent with only systolic dysfunction. Histology confirmed the LVNC phenotype in *Taz^PM^*♂ juveniles, as both prominent LV trabeculations and lack of definitive ventricular apex are observed (Figure S3F). However, unlike adult *Taz^PM^*♂, juvenile *Taz^PM^*♂ hearts do not yet have any detectable cardiac fibrosis, elevated Nppa/Bmp10, or abnormal periostin or lipid deposition evident. Combined, these findings indicate a progressive cardiomyopathy phenotype with chronic LV systolic dysfunction with early onset in juvenile *Taz^PM^*♂ mice and subsequent progressive fibrosis and cardiac lipid dysregulation in aging *Taz^PM^*♂ adults. Thus, the time-course of knockin *Taz^PM^*♂ cardiomyopathy development is different from the *Taz^KO^*♂ deletion model and reinforces the importance of patient mutation-informed models, as *Taz^KO^*♂ juvenile hearts have normal cardiac function and only adult *Taz^KO^*♂ (8 weeks onwards) exhibit cardiac dysfunction [8].

To examine the molecular basis underlying this progressive pathology, we performed temporal protein analysis. Because adult *Taz^PM^*♂ exhibit extensive fibrosis and HF, we first examined profibrotic and cardiac output prognostic biomarkers. Western blotting revealed ectopic induction of profibrotic galectin-3 (Gal3) along with a significant increase in autophagic cathepsin D lysosomal protease isoforms (CathD) in adult *Taz^PM^*♂ ventricles compared to *wt*♂ (Figure 3C and Figure S4). As both Gal3 and CathD can be stress-induced in immune cells and CMs, we confirmed localized upregulation within the adult *Taz^PM^*♂ CMs (Figure 3E,G). Western blotting also revealed a significant reduction in expression of the sarcoplasmic/endoplasmic reticular calcium (Ca^2+^)-ATPase (Serca2a), as well as reduced levels of total phospholamban (Pln), Ser^16^-phosphorylated Pln (pPln^S16^), Thr^286^-phosphorylated Ca^2+^/CaM kinase II isoforms (pCaMKII ^T286^), and Thr^197^-phosphorylated protein kinase A (pPka^Thr197^), in the hearts of adult *Taz^PM^*♂ mice (Figure 3C). These findings suggest the possibility that the massive reduction in total Pln paired with only a small residual pPln expression is insufficient to compensate for the reduced Serca2 expression [27,28]. Besides the role of reduced Serca2 activity in contributing to the HF phenotype of adult *Taz^PM^*♂ mice, the observed changes in pCaMKII and pPKA expression may also contribute to contractile failure via dysregulation of voltage-gated Ca^2+^ and Ca^2+^ release channels on the SR membrane, Pln alteration, or a combination of these [27,28]. Further, mitochondrial Ca^2+^ cycling/load/proton gradient Uncoupling protein-3 (Ucp3) and Ca^2+^ Uniporter (Mcu) are significantly suppressed in adult *Taz^PM^*♂. In accordance with juvenile *Taz^PM^*♂ mildly diminished LV function (see Figure S3), both stress-induced HF biomarkers, Gal3 and CathD, already exhibit some upregulation (Figure 3C). However, the expression profile of Ca^2+^ regulatory proteins in juvenile *Taz^PM^*♂ hearts were distinctly different from that seen in adult *Taz^PM^*♂ hearts (Figure 3C and Figure S4). Unlike in adult mutant hearts, Serca2 and pPln levels were unchanged compared to *wt*♂, while Pln trended higher (Figure 3C). Although pCaMKII, Ucp3, and Mcu remain significantly suppressed, pPka is unaltered in *Taz^PM^*♂ juvenile ventricles (Figure 3C). Thus, the combined effects of an increase in pPln and a decrease in the Pln/Serca2 ratio would be expected to augment SR Ca^2+^ content and improve contractility, contrary to what we observed echocardiographically in juvenile mutant mice. It is possible that the observed change in pCaMKII adversely affects the activities of other Ca^2+^ handling proteins (such as those assessed above), giving rise to reduced LV contractility. It is worth noting, however, that the magnitudes of LV contractile indices were significantly larger in juvenile versus adult mutants, which is consistent with the observation that Serca2 expression levels were unchanged in juvenile mutant hearts whereas they were drastically reduced in adult mutant hearts (Figure 3C). Overall, while expression changes in Ca^2+^ regulatory proteins may be at least partially responsible for the systolic failure of mutant hearts, gaining insight into their functional consequences will require future detailed CM-level studies.

### 2.4. Progressive Metabolic Shift in Surviving Taz^PM^ Male Hearts

As the heart is the most metabolically demanding organ in the body, it needs to constantly produce enormous amounts of ATP [28]. Consequently, HF is generally associated with altered energy metabolism, primarily resulting from decreased ATP generation [28]. As TAZ can catalyze fatty acid exchanges between phospholipids via phospholipid-lysophospholipid transacylation, we examined whether both dysregulated fatty acid and/or accompanying aberrant amino acid metabolism are present in adult and juvenile *Taz^PM^*♂ hearts. As expected, failing adult *Taz^PM^*♂ ventricles exhibit a remarkable reduction in enzymes that regulate fatty acid oxidation, evidenced via heightened degradation and release from phospholipids as acetyl-CoA acyltransferase 2 (Acaa2), acyl-CoA synthetase long-chain 1 (Acsl1), and hydroxyacyl-Coenzyme-A dehydrogenase (Hadh) mitochondrially-targeted enzymes are all significantly reduced (Figure 3H and Figure S4). While Hadh is part of the fatty acid β-oxidation pathway, Acaa2 catalyzes the last step of the oxidation spiral, and Acsl1 is required for long-chain fatty acid uptake and preservation of cardiac output, suggesting wholesale suppression of adult *Taz^PM^*♂ fatty acid metabolism. However, juvenile *Taz^PM^*♂ ventricles expressed unaltered Acaa2/Acsl1/Hadh levels (Figure 3H). Given the reduction in mature CL observed (Figure 1D), and like the aforementioned fatty acid metabolism biomarkers, phospholipase A2 group VI (Pla2g6) is also significantly suppressed in adult *Taz^PM^*♂ but increased in juvenile *Taz^PM^*♂ (Figure 3H). Pla2g6 catalyzes the release of fatty acids from phospholipids, co-localizes with TAZ in the inner mitochondrial membrane (IMM), and may function in CL elimination [29]. In parallel, failing adult *Taz^PM^*♂ ventricles also exhibit a clear reduction in the enzymes that regulate amino acid metabolism. Specifically, lactate dehydrogenase (Ldh), arginase (Arg1), and sodium-coupled neutral amino acid transporter 1 (Slc38a1) are all significantly reduced in adult *Taz^PM^*♂ but were unaltered in juvenile *Taz^PM^*♂ (Figure 3H and Figure S4). The lack of Ldh/Arg1/Slc38a1 suggests adult *Taz^PM^*♂ ventricles may exhibit a dysfunctional Krebs cycle, resulting in reduced amino acids/metabolic intermediates (arginine, glutamine, lactate, pyruvate) and a greater reliance upon glycolysis in adults. Indeed, BTHS patients can present with reduced arginine plasma levels, along with altered glutamine, cystine, proline, and asparagine amino acids [10,30]. Taken together, these results suggest adult *Taz^PM^*♂ have a drastic dysregulation of fatty acid and amino acid metabolism. As a result, a decrease in energy production only occurs during adult advanced cardiomyopathy stages, while juvenile *Taz^PM^*♂ mitochondrial oxidation is unaffected. 

### 2.5. Surviving Taz^PM^♂ Hearts Exhibit Structurally and Functionally Abnormal Mitochondria

**Figure 4:**
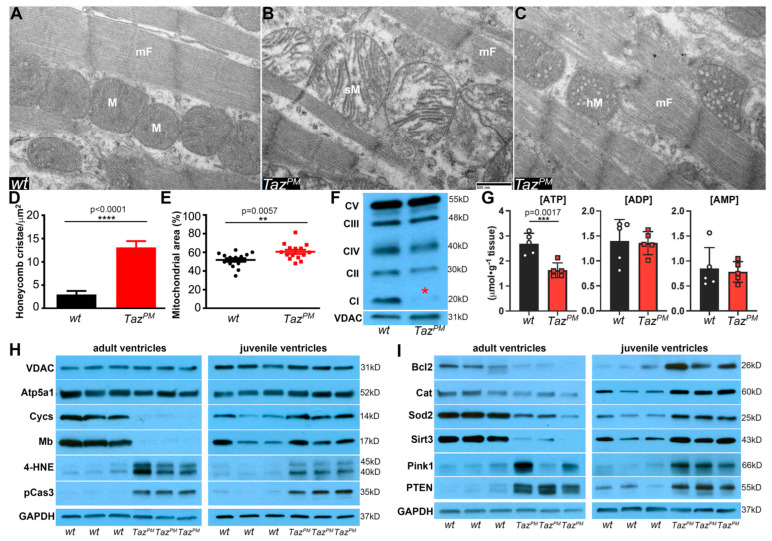
Dysmorphic *Taz^PM^*♂ mitochondrial structure and function is offset via upregulation of cardioprotective pathways. (**A**–**C**), Electron microscopy showing adult *wt*♂ (**A**) and *Taz^PM^*♂ swollen (**B**), and honeycomb (**C**) mitochondrial morphology. (**D**,**E**) Quantification of “honeycomb” mitochondria (**D**) and measurement of the mitochondrial area as % of LV in *wt*♂ (*n* = 6) vs. *Taz^PM^*♂ (*n* = 7) LVs (**E**). (**F**) Western blot image of the individual mitochondrial complexs’ expression in *wt*♂ and *Taz^PM^*♂ mitochondrial fractions probed using an OxPhos rodent antibody cocktail kit, and mitochondrial VDAC as the loading control. Note that C1 (*) levels are severely reduced. (**G**) Adenine nucleotide contents (μmol.g^−1^ tissue) in adult *wt*♂ and *Taz^PM^*♂ ventricles (*n* = 5/genotype). Note, *Taz^PM^*♂ ATP levels were significantly reduced (*p* = 0.0017) while ADP and AMP levels remained unaltered (*p* = ns). (**H**,**I**) Western analysis showing key mitochondrial effector (**H**) and cardioprotective (**I**) expression levels within triplicate *wt*♂ and *Taz^PM^*♂ adult and juvenile ventricles (*n* = 6 per genotype/age). GAPDH was used as a loading control. Quantification and statistical analysis are shown in Supplemental Figure S6. Statistical significance set at ** *p* < 0.01, *** *p* < 0.005 and **** *p* < 0.001. Abbreviations: M, mitochondria; sM, swollen mitochondria; hD, honeycomb mitochondria; mF, myofibrils. Scale bars, (**A**–**C**) = 500 nm.

As mitochondrial defects are predominant in BTHS, TAZ is present in all mitochondria, and TAZ itself impacts many aspects of mitochondrial structure-function, we examined how the loss of *Taz^PM^*♂ transacylase activity affected mitochondrial morphogenesis, oxidative phosphorylation (OxPhos), and, above all, the production of ATP. First, as CL is required for mitochondrial structural integrity and cristae morphology [5,7,31], we examined mitochondrial structure and number. Ultrastructural transmission electron microscopy revealed surviving adult *Taz^PM^*♂ ventricles contain a mixture of abnormal mitochondria, including swollen mitochondria with widened, collapsed cristae (Figure 4B) and a significant increase in normal-sized but honeycomb-like mitochondria containing bundled hollow cristae (Figure 4C,D), resembling those seen in BTHS [5,7,31]. However, we did not find any vacuolated mitochondria with sparse/absent cristae, suggesting anomalous cristae morphogenesis rather than a lack of formation or degradation. As there are conflicting reports of mitochondrial content in *Taz* shRNA knockdown and knockout mice [8,17,18,19,20], we quantified mitochondrial area as % of the entire LV area. There is a small but significant increase in the area occupied by mitochondria in *Taz^PM^*♂ LV myocytes compared to *wt*♂ littermates (Figure 4E). However, none of the adult *Taz^PM^*♂ hearts displayed any contractile apparatus thick- or thin-filament changes. Thus, most *Taz^PM^*♂ cardiac mitochondria exhibit morphological abnormalities. 

Second, as CL is associated with the OxPhos mitochondrial ETC supercomplexes and is required for their clustering/proper enzymatic activity/stability [2,9,11,13], we assessed individual ETC subunit levels in surviving adult *Taz^PM^*♂ mitochondria. We found that *Taz^PM^*♂ exhibit reduced expression of Complex I (NADH: ubiquinone oxidoreductase subunit B8, NDUFB8), but Complex II (Succinate dehydrogenase, SDH), Complex III (Ubiquinol-cytochrome c reductase core protein 2, UQCRC2), Complex IV (Cytochrome c oxidase I, mitochondrial, mt-Co1), and Complex V (ATP synthase, H^+^ transporting, mitochondrial F1 complex, alpha subunit) levels were all unchanged (Figure 4F). The primary function of the ETC is to generate an electrochemical gradient that can be used to drive ATP synthesis [2] and as CL is the main membrane phospholipid required for Complex 1 activity [9,11,12], thus reduced Complex I is likely to result in a reduction of proton-driven ATP synthesis in *Taz^PM^*♂ ventricular mitochondria. 

Third, as the central function of mitochondria is to provide energy via ATP production [27,28], we directly measured both oxidized and reduced nicotinamide adenine dinucleotide (NAD^+^, NADH) and coupled adenine nucleotide (ATP, ADP, and AMP) levels. Using ultra-performance liquid chromatography, we found adult *Taz^PM^*♂ NAD^+^ levels trend ~14% lower, but that NADH was unaffected in mutant ventricles compared to *wt*♂ littermates (Figure S5A). As NAD^+^/NADH dictates how effectively the cell can produce ATP, a lower ratio could indicate unfavorable conditions for oxidative reactions in adult *Taz^PM^*♂ ventricles. Critically, adult *Taz^PM^*♂ ATP levels were significantly reduced ~40% while ADP and AMP levels remained unaltered (Figure 4G). Further, despite the total adenine nucleotide pool (ATP + ADP + AMP) usually being tightly regulated [32], it is also discernibly reduced ~22% in mutants compared to *wt*♂, as well as the ATP/ADP ratio, an indicator of available free energy, being reduced (Figure S5B). This ~40% drop in adult *Taz^PM^*♂ ATP levels are comparable to the ~30% drop observed in most patient and animal HF models [28]. However, despite a systemic absence of acyltransferase function, juvenile *Taz^PM^*♂ NAD^+^/NAHD levels, as well as ATP/ADP/AMP levels, were unaffected (Figure S5C,D), reinforcing the progressive cardiomyopathy phenotype and suggesting either a cumulative deterioration or a subsequent failure of a juvenilely-active compensatory mechanism.

Finally, to gain a more detailed understanding of the progressive effects of the *Taz^D75H^* point mutation within mitochondria, we examined key mitochondrial effectors. In agreement with our TEM mitochondrial area and ETC Western findings, adult and juvenile *Taz^PM^*♂ voltage-dependent anion channel (VDAC) levels were unaffected (Figure 4H and Figure S6). Reflecting the drop in only adult *Taz^PM^*♂ ATP levels, mitochondrial ATP synthase (Atp5a1) is reduced in *Taz^PM^*♂ adults but increased in *Taz^PM^*♂ juvenile ventricles. Moreover, hemeprotein cytochrome c (Cycs) is significantly suppressed in adults but upregulated in juvenile *Taz^PM^*♂. Hemeprotein myoglobin (Mb) is also significantly reduced in *Taz^PM^*♂ adults but unchanged in juvenile *Taz^PM^*♂ (Figure 4H and Figure S6). We reasoned that increased Cycs and unaltered Mb likely function as juvenile cardioprotective antioxidants, while complete suppression of Cycs/Mb in adult *Taz^PM^*♂ ventricles may foster oxidative stress and/or caspase activation. Accordingly, we measured levels of the lipid peroxidation/oxidative stress biomarker 4-hydroxynonenal (4-HNE), which we found to be significantly elevated in both adult *Taz^PM^*♂ and juvenile *Taz^PM^*♂ ventricles (Figure 4I and Figure S6). Although 4-HNE is toxic, it can also control physiological redox signaling and modulate membrane potentials [33]. Correspondingly, pro-caspase3 (pCas3) levels were significantly elevated in both juvenile and adult *Taz^PM^*♂ ventricles (Figure 4H and Figure S6), but cleaved caspase3 levels were unaffected. Together, these results indicate that pCasp3 induction failed to elicit a *Taz^PM^*♂ apoptotic response, which is consistent with our observation that TUNEL is negative in *Taz^PM^*♂ hearts (Figure S2I). Rather, increased pCasp3 may reflect this protein’s known function in pro-survival cell growth, autophagy, and homeostatic maintenance in normal cells. We, therefore, assessed levels of additional proteins that may preserve *Taz^PM^*♂ juvenile heart integrity. We detected significantly increased levels of cardioprotective mitochondrial proteins B-cell lymphoma-2 (Bcl2), antioxidant catalase (Cat), ROS scavenger superoxide dismutase-2 (Sod2), and NAD^+^-dependent deacetylase mitochondrial sirtuin-3 upregulation (Sirt3). However, in adult *Taz^PM^*♂ ventricles, Bcl2, Cat, Sod2, and Sirt3 levels are all reduced. Moreover, cardioprotective PTEN-induced kinase-1 (Pink1) and its inducer phosphatase and tensin homolog deleted on chromosome 10 (PTEN) are upregulated in *Taz^PM^*♂ ventricles (Figure 4I). Hence, numerous cardioprotective effectors are boosted in juvenile *Taz^PM^*♂ ventricles, while PTEN/Pink1 and 4-HNE/pCasp3 pro-survival effectors are constitutively induced in *Taz^PM^*♂, thereby offsetting elevated Ca^2+^/oxidative stress and diminished ATP-mediated pathological abnormalities that could trigger apoptosis. 

### 2.6. Autophagy-Mediated Senescence

**Figure 5:**
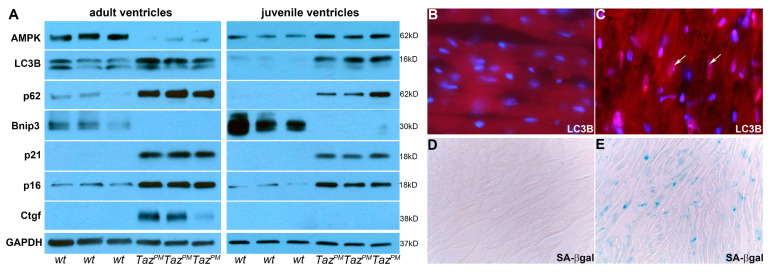
Induction of autophagic and senescent adaptive stress responses in surviving *Taz^PM^*♂ hearts. (**A**) Western analysis showing key autophagy and senescence biomarker expression levels within triplicate *wt*♂ and *Taz^PM^*♂ adult and juvenile ventricles (*n* = 6 per genotype/age). GAPDH was used as a loading control. Quantification and statistical analysis are shown in Supplemental Figure S7. (**B**,**C**) Immunofluorescence to detect autophagic protein *LC3B* (arrows, red fluorescence) upregulation in juvenile *Taz^PM^*♂ (**C**) but not *wt*♂ (**B**) ventricles. Nuclei are marked by DAPI (blue fluorescence). (**B**,**C**) were magnified ×630. (**D**,**E**) Representative image of senescence-associated βgal (SA-βgal) staining in adult *wt*♂ (**D**) and *Taz^PM^*♂ (**E**) ventricles. Note, only *Taz^PM^*♂ LVs are positive for SA-βgal (blue). (**D**,**E**) were magnified ×400.

Given that undersized juvenile *Taz^PM^*♂ hearts demonstrate upregulation of numerous cardioprotective pathways, we examined whether these stress responses were due to transient induction of pro-survival reprogramming [34,35]. Because adult *Taz^PM^*♂ hearts progressively lose functional capacity and downregulate several cardioprotective pathways, we examined whether worsening *Taz^PM^*♂ cardiomyopathy was due to a subsequent loss of pro-survival autophagy, mitophagy, and/or upregulation of pathological cellular senescent reprogramming. We found that the autophagy-inducing AMP-activated protein kinase sensor (AMPK), autophagy marker microtubule-associated proteins 1A/1B light chain 3B (both LC3B isoforms LC3-I and LC3-II), and autophagy receptor sequestosome-1 (p62) levels were all significantly accumulated in *Taz^PM^*♂ juvenile ventricles (Figure 5A and Figure S7). Immunofluorescence confirmed characteristically activated autophagic punctate LC3B in *Taz^PM^*♂ juveniles, compared to *wt*♂ (Figure 5B,C). Conversely, in adult *Taz^PM^*♂ ventricles, AMPK levels are significantly reduced, but the cytoplasmic LC3-I isoform and p62 levels remain elevated (Figure 5A). Consequently, along with the progressive upregulation of autophagic-associated CathD (see Figure 3C), these data establish that there is an initial adaptive survival-oriented AMPK response in *Taz^PM^*♂ juveniles. However, loss of AMPK in adult *Taz^PM^*♂ ventricles, despite LC3-I/p62 upregulation, is sufficient to cause metabolic dysfunction and result in progressive cardiomyopathy. Concordantly, unaltered membrane/autophagosome-bound LC3-II levels and isolated p62 accumulation are typically interpreted as reduced autophagosome formation and inhibition of autophagy [34,35]. Further, expression of mitochondrial death and mitophagy marker BCL2-interacting protein 3 (Bnip3), is absent in both juvenile and adult *Taz^PM^*♂ (Figure 5A), indicating mutant cardiac mitochondria fail to undergo Bcl2/Bnip3-associated mitophagy. Indeed, the inability to undergo mitophagy can itself result in lipid accumulation and the exacerbation of cardiomyopathy [36].

As metabolic dysfunction allied with aberrant mitochondrial dynamics can initiate cellular senescence [33,37], and as autophagy can both suppress and promote cardiac senescence [36,37], we examined senescent cell cycle arrest, apoptosis resistance, and senescence-associated secretory phenotype biomarkers. Significantly, the cyclin-dependent kinase inhibitor 1A (p21) marker of senescent transition is ectopically induced in both juvenile and adult *Taz^PM^*♂ ventricles. Further, cyclin-dependent kinase inhibitor 2A (p16) levels are also significantly elevated in both juvenile and adult *Taz^PM^*♂ ventricles (Figure 5A and Figure S7). As p16 is required to maintain the senescent phenotype and acts as a point of no return [38], this indicates both juvenile and adult *Taz^PM^*♂ ventricles exhibit enhanced senescence signaling. In conjunction with the increased expression levels of these cell cycle inhibitors, late senescence-associated β-galactosidase was only detected in adult *Taz^PM^*♂ but not *wt*♂ ventricles (Figure 5E). Expression levels of terminal senescence-associated secretory phenotype markers, namely β-galactoside-binding proinflammatory Gal3 (see Figure 3C) and proinflammatory and profibrotic connective tissue growth factor (Ctgf), are ectopically induced in *Taz^PM^*♂ adult hearts (Figure 5A). In contrast, Gal3 is only weakly elevated (Figure 3C), and Ctgf is absent in *Taz^PM^*♂ juveniles (Figure 5A). Likewise, the senescence inhibitor Sirt3 is also elevated in juveniles and reduced in adult *Taz^PM^*♂ (see Figure 4I). To determine whether protein level changes are due to transcriptional and/or post-transcriptional regulation, we used qPCR to measure relative mRNA levels. Only *p62* mRNA levels were significantly transcriptionally elevated in both adult and juvenile *Taz^PM^*♂ ventricles, while *p21* was upregulated in adult *Taz^PM^*♂ and *Prkaa1* (AMPK catalytic α subunit) was suppressed in juvenile *Taz^PM^*♂ ventricles (Figure S8). However, *Prkaa2* (AMPK catalytic *β* subunit), *Lc3b*, *Bnip3*, and *p16* were unaffected at both ages (Figure S8), indicating that *Taz^PM^*♂ altered protein levels did not occur at the transcriptional level. Collectively, these data suggest that pro-survival AMPK/LC3B/p62-mediated autophagy and pathological p21/p16-mediated senescence are both induced in juvenile *Taz^PM^*♂ ventricles, but that only pathological p21/p16-mediated terminal senescence remains activated in adult *Taz^PM^*♂ ventricles, thereby directing the severity and chronic progression of adult *Taz^PM^*♂ cardiac pathophysiology. 

### 2.7. Biphasic p53 Pathway Hyperactivation

**Figure 6:**
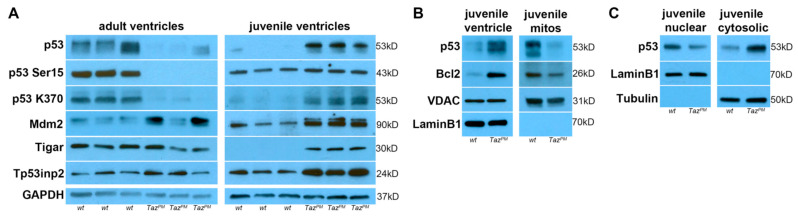
Transient p53 pathway activation in surviving *Taz^PM^*♂ hearts. (**A**) Western analysis showing p53 and key p53 effector expression levels within triplicate *wt*♂ and *Taz^PM^*♂ adult and juvenile ventricles (*n* = 6/genotype/age). GAPDH was used as a loading control. (**B**) Representative western analysis of whole cell (juvenile ventricle) and isolated mitochondrial (juvenile mitos) fractions from juvenile *wt*♂ and *Taz^PM^*♂ ventricles (*n* = 3/genotype/isolation method). Total p53 and Bcl2 are upregulated in *Taz^PM^*♂ whole cell fraction but are suppressed in isolated *Taz^PM^*♂ mitochondrial fractions. VDAC was used as a loading control and to verify mitochondrial enrichment. LamininB1 was used as a loading and purity control since nuclear LamininB1 is only present in whole cell fractions. (**C**) Representative western of nuclear and cytosolic fractions from juvenile *wt*♂ and *Taz^PM^*♂ ventricles (*n* = 3/genotype/isolation method). Total p53 is slightly down in *Taz^PM^*♂ nuclear but is robustly upregulated in *Taz^PM^*♂ cytosolic fraction. Nuclear LamininB1 was used as a loading and nuclear fraction purity control, and cytosolic Tubulin was used as a loading and cytosolic fraction purity control. Quantification and statistical analysis are shown in Supplemental Figure S7.

In addition to its canonical basal or low-stress survival and tumor suppressive functions, p53 is also a key component of the metabolic stress response machinery, as it can control fatty acid oxidation sensors, regulate both autophagy and senescence, decrease glycolysis, and increase oxidative phosphorylation [39,40]. As we found p53 downstream targets AMPK/Bcl2/Bnip3/Casp3/CathD/LC3B/LDH/p21/p62/PTEN/Serca2/Sirt3 are all mis-expressed in *Taz^PM^*♂ ventricles (see Figure 3, Figure 4 and Figure 5), we examined the p53 pathway directly. Significantly, total p53 protein levels are dramatically increased in juveniles but sharply suppressed in adult *Taz^PM^*♂ ventricles (Figure 6A and Figure S7), suggesting p53 is stabilized and p53 effectors are likely to be transiently activated in juveniles but not maintained in failing adult *Taz^PM^*♂ hearts. As p53 undergoes various post-translational modifications that alter the selectivity of target genes and ensure p53 signaling is only transiently activated [40,41], we examined both phosphorylation and acetylation status and p53-associated metabolism targets. Both p53 phosphorylation at Ser15 (an indicator of p53 stabilization) and acetylation at K370 (facilitates p53 activation) are significantly increased in juvenile *Taz^PM^*♂ ventricles (Figure 6A). However, in adult *Taz^PM^*♂, p53^Ser15^ phosphorylation is completely abolished and p53^K370^ acetylation is drastically reduced, indicating DNA binding/transcriptional activity are severely diminished in only adult mutants. Further, the level of p53’s major antagonist, mouse double minute-2 (Mdm2), is significantly increased in juvenile *Taz^PM^*♂ and trends upwards in adult *Taz^PM^*♂. The p53-induced Mdm2 alternative transcripts can be triggered by stress and may amplify p53 protective mechanisms [41]. Consistent with this phenomenon, we detected an additional Mdm2 isoform in *Taz^PM^*♂ but not in *wt*♂ juveniles. Likewise, downstream p53-induced glycolysis and apoptosis regulator (Tigar) and tumor protein p53 inducible nuclear protein-2 (Tp53inp2) cardioprotective and autophagic effectors are present at higher levels in juvenile *Taz^PM^*♂ (Figure 6A and Figure S7). However, Tigar, and Tp53inp2 levels are statistically unaltered in adult *Taz^PM^*♂. Hence, the p53 signaling pathway is activated in juvenile *Taz^PM^*♂ but largely stifled in surviving adult *Taz^PM^*♂ hearts that undergo HF. qPCR analysis revealed adult and juvenile *Taz^PM^*♂ *p53*, *Mdm2*, *Tigar*, and *Tp53inp2* mRNA levels were all unchanged (Figure S8), indicating post-transcriptional mechanisms (i.e., altered protein translation rates, turnover, intracellular location, and/or molecular association of the protein products) likely underlie p53 pathway hyperactivity.

Although basal p53 is mainly localized in the cytoplasm in unstressed cells, stress-activated and post-translationally modified increased p53 can be translocated into multiple cellular compartments, including the nucleus and IMM [42,43]. To determine if juvenile *Taz^PM^*♂-mediated p53 transient upregulation is present in mutant mitochondria, we isolated juvenile ventricular mitochondria and compared their p53 levels to juvenile ventricular total protein levels. As expected, both total p53 and Bcl2 levels are significantly higher in *Taz^PM^*♂ juvenile ventricle whole protein samples compared to *wt*♂ littermates. Also, both total p53 and Bcl2 levels are significantly lower in *Taz^PM^*♂ juvenile isolated mitochondrial protein samples compared to *wt*♂ (Figure 6B). This demonstrates that *Taz^PM^*♂-mediated p53 and Bcl2 upregulation is largely absent from juvenile mutant heart mitochondria. As cytoplasmic and nuclear p53 can both independently respond to stress via transcription-dependent and -independent mechanisms, both of which are regulated by posttranslational modifications [43], we examined if juvenile *Taz^PM^*♂-mediated p53 upregulation is present in the nucleus, cytoplasm, or both. Significantly, total p53 levels are robustly increased in the *Taz^PM^*♂ cytoplasm but reduced in *Taz^PM^*♂ nuclear samples (Figure 6C). Combined, these data indicate that juvenile *Taz^PM^*♂-mediated total p53 upregulation is mainly confined to the mutant cytoplasm and is largely excluded from mutant mitochondria, resulting in a lack of mutant mitochondrial apoptosis. 

### 2.8. Genotype-Phenotype-Specific p53-Related Reprogramming

As many *TAZ* mutations result in the production of mutant TAZ proteins with little or no function [16,17,19,20] rather than a complete absence of TAZ protein, we compared the proteome of systemic *Taz* knockout hearts (*Taz^KO^*) that completely lack Taz protein [8] with our BTHS patient-specific mouse *Taz^PM^*♂ model that expresses a stable mutant Taz^D75H^ protein. *Taz^KO^* mice recapitulate many of the key clinical features of BTHS, including neonatal lethality, impaired growth, suppressed CL, dilated cardiomyopathy, and skeletal myopathy [8,21]. Significantly, whilst most differentially expressed *Taz^PM^*♂ biomarkers are comparably altered and/or trend similar in adult *Taz^KO^* hearts (Figure S9B), p53 and several p53-dependent targets are not differentially misexpressed in *Taz^KO^* hearts. Notably, p53 levels, which are significantly reduced in adult *Taz^PM^*♂ hearts remain unchanged in *Taz^KO^* despite the p53 negative regulator Mdm2 being reduced in *Taz^KO^* (Figure S9A). Further, the p53 effectors Tp53inp2 and Tigar are lower in *Taz^KO^* hearts but are either unaltered or elevated in *Taz^PM^*♂ hearts. PTEN, which is directly regulated via a p53 binding element [44], is strongly increased in *Taz^PM^*♂ but unaltered in *Taz^KO^*. Moreover, p53-dependent AMPK and LC3B levels are unaltered in *Taz^KO^* but are significantly reduced in surviving *Taz^PM^*♂ hearts, while the p53-independent p62 autophagy receptor expression is similarly upregulated in both *Taz^PM^*♂ and *Taz^KO^* (Figure S9B and Figure 5A). Further, the terminal senescent marker p16 is unaltered in *Taz^KO^* hearts, in contrast to being constitutively upregulated in *Taz^PM^*♂. Combined, these data suggest that most of these HF, mitochondrial, and metabolism biomarkers are commonly mis-expressed in both loss-of-function and targeted knock-in *Taz* mutant allele models, but that p53 pathway dysregulation and autophagy-mediated senescence are mainly observed in our point-mutant knock-in BTHS model. A recent study examining single nuclei gene expression in *Taz^KO^* hearts also did not reveal p53 pathway dysregulation [45], suggesting the presence of an enzymatically deficient Taz^D75H^ protein is itself sufficient to elicit a unique response. Further studies will be necessary to determine whether these p53-mediated effects occur independently of transacylase activity and/or whether the Taz^D75H^ protein actively excludes p53 from mitochondria, underscoring the importance of studying patient-derived missense mutations.

## 3. Discussion

Although BTHS patients exhibit mutations along the entire *TAZ* gene [6,16], of the current 57 BTHS mutations listed within the *TAZ* acyltransferase domain [https://www.barthsyndrome.org, accessed 4 March 2024], only 38 are predicted to be pathogenic, 13 are variants of uncertain significance, and 6 are benign. Most variants are either single missense, nonsense, or frameshift mutations, and BTHS alleles frequently express mutant proteins [16,17,18,19]. Our limited understanding of the molecular consequences specific to distinct *TAZ* mutations is an obstacle to progress toward defining genotype-phenotype correlations and improving outcomes for BTHS patients. To overcome this hurdle, we have generated the first patient-tailored BTHS knock-in mutant mouse model (*Taz^PM^*) harboring a patient-derived point mutation in the critical *Taz* acyltransferase domain required to remodel MLCL to mature CL. Significantly, the *Taz^PM^*♂ mutants recapitulate the major BTHS phenotypes. Notably, the *Taz^PM^* allele generates a stable Taz^D75H^ mutant protein expressed at equivalent levels to wild-type Taz, enabling us to separate the production of Taz protein from its enzymatic function. Consequently, despite the lack of *Taz^PM^*♂ acyltransferase activity and insufficient CL, Taz^D75H^ protein mitochondrial localization, membrane anchoring, and unique hydrophilic and protein import domains all remain intact [9,13,46]. Indeed, non-mutant *TAZ* mRNAs undergo alternative splicing, and there are naturally occurring isoforms that lack transacylase activity, yet these proteins also localize to mitochondria, and their function remains unknown [6,17]. Thus, the Taz^D75H^ protein may still play a functional role itself in the structural integrity, bioenergetic function and protein-protein interactions within *Taz^PM^*♂ mitochondria [11,12,13]. This may partially explain our unique phenotype-genotype biomarker data and why, despite exhibiting similar structural/functional anomalies, the adult *Taz^PM^*♂ hearts contain normal mitochondrial numbers and fail to exhibit apoptosis as observed in knockdown *Taz^KD^* and deficient *Taz^KO^* mice, respectively [8,20]. Moreover, combined CL reduction and absent Cycs likely prevent the release of key proapoptotic effectors in *Taz^PM^*♂ hearts [47]. Thus, while previous transgenic mouse models either lack [8,18,19] or express diminished wild-type Taz protein [20] or exhibit independent doxycycline-mediated OxPhos suppression [46], our *Taz^PM^* allele provides, to the best of our knowledge, the first animal model that faithfully recapitulates the genomic origin of BTHS and serves as the first in vivo BTHS model expressing a mutant protein.

In keeping with cardiomyopathy being the most common BTHS clinical feature [4,5,7,8,15,17,18,19,20], *Taz^PM^*♂ display fully penetrant heart phenotypes throughout life. Clinical history reveals BTHS individuals can exhibit systolic dysfunction that changes over time, with neonatal cardiac function being severely compromised, followed by LV dilation regression and systolic function recovery, and finally entry into a stable phase in which LVEF is preserved (at ≈50%) prior to eventual HF [48]. Reflecting this continuum of BTHS pathology, only ~50% of *Taz^PM^*♂ mutants survive to adulthood, and all the surviving *Taz^PM^*♂ mutants exhibit a progressive cardiomyopathy phenotype, with mildly affected LV function in *Taz^PM^*♂ juvenile diminutive LVNC hearts that transition into a deteriorating end-stage HF in undersized *Taz^PM^*♂ adult LVNC hearts. Only adult *Taz^PM^*♂ hearts exhibit fibrotic extracellular matrix (ECM) deposition and significant steatosis that likely contribute to additional LV contractile dysfunction and premature lethality seen in adult *Taz^PM^*♂ hearts. As matricellular Periostin is an early TGFβ-induced pro-fibrotic marker whose elevation is associated with both patient and mice HF [24], this upregulation of ECM synthesis indicates reactive interstitial fibrosis. Moreover, as periostin can modulate anti-fibrotic p53 expression [49] and adult *Taz^PM^*♂ fibrosis coincides with the loss of p53 hyperactivation, proliferative juvenile cardiac fibroblasts likely are released to undergo transformation and generate the adult mutant robust fibrotic response [50]. Further, this functional transition coincides with large-scale enzymatic changes in adult *Taz^PM^*♂ that indicate subsequent suppression of fatty acid and amino acid metabolism, absent Cycs/Mb-mediated oxygen transport, reduced OxPhos, and an increased reliance upon glycolysis, along with robust dysregulation of Serca2a-mediated cardiac contractility. Indeed, a ~35% decrease in Serca2 is sufficient to cause decreased CM contractility and sarcoplasmic reticulum Ca^2+^ load [27]. Thus, *Taz^PM^*♂-mediated Serca2a suppression (despite a massive reduction in Serca2-inhibiting PLN), combined with a lack of Sarcalumenin Ca^2+^ buffering, decreases SR Ca^2+^ load in *Taz^PM^*♂ adult hearts reducing LV contractility. Moreover, along with multifunctional pCaMKII signaling, *Taz^PM^*♂ mitochondrial Ucp3/Mcu levels are also suppressed, suggesting further dysregulation of intracellular Ca^2+^ homeostasis/Ca^2+^ uptake and uncoupling of *Taz^PM^*♂ IMM potential. As Ucp3 abundance is directly correlated with the degree of fatty oxidation, is frequently downregulated in human HF, and reduced Ucp3 levels correlate with an increased incidence of LV diastolic dysfunction [51], the reduced Ucp3 levels reinforce systemic *Taz^PM^*♂ cardiac dysfunction. Further, as the Mcu pore is responsible for the majority of Ca^2+^ uptake and ATP production [52], robust adult *Taz^PM^*♂ suppression of Mcu suggests additional altered IMM Ca^2+^ uptake, a reduced bioenergetic reserve capacity and/or loss of cardioprotective regulation of cytosolic Ca^2+^ in mutant cardiomyocytes.

Metabolic processes vary markedly in the hearts of *Taz^PM^*♂ mice. In *Taz^PM^*♂ juveniles, fatty acid and amino acid metabolism enzymes and ATP levels were initially unchanged versus age-matched *wt*♂ mice. In contrast, adult *Taz^PM^*♂ hearts exhibit robust suppression of fatty acid and amino acid metabolism enzymes, along with a consequential ~40% drop in ATP content, resulting in severe systolic dysfunction and HF. Allied to this, several mitochondrial cardioprotective and alternative oxidation pathway effectors (Bcl2/Cat/Sod2/Sirt3/Pink1/PTEN) that were induced in the less affected juvenile *Taz^PM^*♂ hearts are absent in deteriorating *Taz^PM^*♂ adults. Underlying this transition to a more severe adult cardiomyopathy, we demonstrated that suppressed *Taz^PM^*♂ mitochondrial function may be due, in part, to reduced ETC Complex I, resulting in the observed lower production of mitochondrial ATP and hence a reliance upon enhanced glycolysis. As CL interacts directly with Complex I and is critical for the formation and stability of respiratory supercomplexes [2,12,53], the lack of Taz^D75H^ acyltransferase enzyme activity and decreased CL are chiefly responsible for reduced adult mutant ATP reduction. Subsequently, adult *Taz^PM^*♂ hearts are unable to use alternative energy substrates (i.e., amino acids) nor rely on adaptive glycolysis to compensate for the overall decrease in ATP supply. Whether this is a consequence of normal development as young highly glycolytic hearts mature into OxPhos-dependent adult hearts [54,55,56], the metabolic and contractility differences that occur with age, or perhaps juvenile hearts are better able to tolerate combined glycolysis/oxidative metabolism, is unknown. Additionally, end-stage pediatric HF adaptive mechanisms to preserve CL content may be intact to a greater degree than those seen in adult HF [57]. Further studies will be required to determine whether changes in these essential metabolic and contractility factors [58] play the same roles during development and the initiation and progression of cardiomyopathy. 

We also demonstrate that the ATP, ATP/ADP ratio, and total adenine nucleotide pool levels were all diminished in *Taz^PM^*♂ adult hearts, indicating the free energy of ATP hydrolysis (the energy available to drive cardiac output [32]) is lower in surviving adult *Taz^PM^*♂. As cardiac ATP is required for multiple processes, including ion transport, muscle contraction, cardiac impulse propagation, substrate phosphorylation, and chemical synthesis [27,28,32], this lower energy state likely directly underlies the myriad of adult cardiac phenotypes and consequential inability to maintain sufficient adult mutant cardiac output. Reduced ATP production and a lower metabolic reserve may also help explain the *Taz^PM^*♂ mild-to-severe phenotype transition, as contractile apparatus cross-bridge cycling and Serca activity are major cellular ATP consumers [59], thereby placing an extra energetic burden upon adult *Taz^PM^*♂ mitochondria. Thus, contractile dysfunction and the inability to attain a normal lifespan are likely secondarily related to ECM remodeling/ectopic lipid deposition and primarily due to OxPhos deficiency, a loss of metabolic flexibility, and mitochondrial cardioprotection, resulting in a predominant lack of energy production in adult *Taz^PM^*♂ cardiomyocytes [3].

Beyond *Taz^PM^*♂ reduced ATP levels and a progressive cardiomyopathy phenotype, one of the most striking findings was the *Taz^PM^*♂ mild-to-severe cardiac transition, along with the onset of adult fibrosis/lipid deposition and ensuing HF. Significantly, this occurred coincident with a biphasic switch from a combined pro-survival AMPK/LC3B-mediated autophagy and stress-activated p21/p16-mediated senescence phenotype in *Taz^PM^*♂ juveniles to a persistent pathological p21/p16-mediated senescence with an accompanying senescence-associated secretory phenotype (SASP) in *Taz^PM^*♂ adults. Furthermore, this shift was accompanied by the loss of juvenile *Taz^PM^*♂ hyperactive p53 signaling within the subsequent failing adult *Taz^PM^*♂ hearts. As *Taz^PM^*♂ juvenile ATP levels are unaffected but p53 and AMPK levels are already amplified, this suggests p53 drives AMPK-mediated autophagy [60] and together they adjust the cellular mitochondrial load to sustain sufficient cardiac output/viability in the mildly affected juvenile *Taz^PM^*♂ hearts. Future temporal studies addressing altered autophagic flux and identification of degradative activity of specific pathways that directly drive the *Taz^PM^*♂ mild-to-severe cardiac transition will be informative. As p53^K370^ acetylation blocks the interaction of p53 with its repressor, Mdm2, directly resulting in p53 activation regardless of its phosphorylation status [61], concomitant upregulation of Mdm2 alongside p53/p53K370/Tigar/Tp53inp2 in *Taz^PM^*♂ juveniles likely fine-tunes hyperactive p53s’ metabolic protective actions. As p53 upregulation is largely absent from juvenile Taz^PM^♂ mitochondria, wherein mitochondrial p53 accumulation normally triggers membrane permeabilization/apoptosis [43], this may help explain why hyperactive p53 plays a cell adaptive response and why *Taz^PM^*♂ hearts do not exhibit apoptosis. Significantly, although both juvenile and adult *Taz^PM^*♂ hearts exhibit a senescent phenotype (as p21/p16 senescence regulators are continually induced), only adult *Taz^PM^*♂ exhibit maladaptive SASP and express late-stage senescence-associated β-gal and Ctgf, signifying irreversible adult senescence [37,38]. Indeed, autophagy inhibition itself can trigger terminal senescence via inhibiting p16 degradation [35,62,63] resulting in chronic resistance to apoptotic and/or growth stimuli as well as paracrine secretion of proinflammatory cytokines, chemokines, growth factors, and profibrotic metalloproteinases, leading to widespread maladaptive remodeling.

BTHS clinical variability is thought to be attributable to a combination of modifying environmental effects and modifier-gene factors [7,10,16,19,64] Therefore, we compared *Taz^PM^*♂ and *Taz^KO^*♂ hearts to better understand whether TAZ has important roles beyond CL maturation. Our analysis revealed that AMPK, hyperactive p53, and p53 pathway activation are uniquely dysregulated in the patient-tailored knock-in *Taz^PM^*♂ mutant mouse model, as opposed to *Taz* deletion/knockdown models [8,18,19,20,45]. This suggests p53 hyperactivation may be a compensatory mechanism that initially protects from Taz^D75H^ loss of function (i.e., altered mitochondrial lipid composition/lack of CL phospholipid) but then eventually fails, leading to cardiomyopathic progression and adult HF. Whether p53 hyperactivation is triggered by the presence of the dysfunctional Taz^D75H^ mutant protein or anomalous protein–protein interactions is presently unknown but highlights the importance of using patient-tailored mouse mutant models to study human syndromes. Although little is known about p53 within BTHS, p53 can cooperate with Sirt’s to regulate CL biosynthesis and reduce oxidative stress in CMs [65,66]. Also, Zebrafish *exon3 Tafazzin* mutants, which recapitulate some features of BTHS, similarly exhibit *p53/Mdm2* mRNA upregulation [16]. Likewise, a BTHS cardiac transplant patient who developed post-transplant lymphoproliferation, contained p53-deficent lymph nodes [67]. Although *TAZ* dysregulation in cancer has not been thoroughly investigated, TAZ is often mis-expressed in p53-dysregulated tumors [68]. Many studies have demonstrated that p53 can directly regulate cardiomyocyte metabolic, oxidative stress, autophagic, senescence, and apoptotic pathways, and p53 can also rheostat cardiac myofibroblast remodeling. Thus, future cardiac-restricted and lineage-specific transgenic approaches will be required to determine which specific p53 targets are temporally dysregulated, as well as where and how juvenile p53 pathway hyperactivation prevents HF that is eventually observed in adult mutants.

Our results provide new insights into the AMPK/p53-mediated transition of juvenile-to-adult heart *Taz^PM^* phenotypes and the mechanism underlying metabolic reprogramming linked to adult BTHS heart failure. Future analysis of p53’s role in BTHS may help identify potential dysfunctional pathways that could offer therapeutic approaches for BTHS patients. Moreover, we have demonstrated that the *Taz^PM^* point mutant protein may have additional effects on mitochondria, gene expression, and cardiac function beyond acyltransferase activity, further distinguishing itself from existing mouse models. Thus, the *Taz^PM^* mouse model will provide an invaluable tool for highly translatable studies, enabling researchers to track progressive disease development, differentiate heterogeneous and intermittent organ defects, perform genotype-phenotype comparisons, and biochemically examine the non-transacylase functions of TAZ. Importantly, the *Taz^PM^* mouse model offers a clinically relevant platform for the in vivo interrogation of potential biomarkers and experimental therapeutic approaches for BTHS. Finally, a potential weakness in this model is that there is significant attrition neonatally. Thus, the analysis presented here reflects the survivors of the *Taz^PM^* point mutation and may be biased in the adult phenotype and gene expression analysis. This is also observed in human disease. Future studies will be required to address these aspects and the Taz^D75H^ protein’s impact on cardiac development.

## 4. Materials and Methods

### 4.1. Sex as a Biological Variable

Our study exclusively examined male mice, as structural cardiomyopathy was not present in female mutants. It is unknown whether the findings are relevant for female mice.

### 4.2. Animal Models

*Tafazzin* (MGI:109626) knock-in mice with a pathogenic mutation (*D75H*: G>C substitution within Asp223 in coding exon 2) were generated using the *CRISPR/Cas9* gene editing system [69]. A novel variant c.223 mutation position in the human *TAFAZZIN* (OMIM:300394) gene is located at the same position in the mouse *Taz* gene (mutation info can be viewed at (www.barthsyndrome.org/research/tafazzindatabase.html, accessed 4 March 2024). *Taz* mRNA and partially complementary single guide RNAs were microinjected into fertilized embryos of C57BL/6J mice, and founder mice were generated (Sage Labs). Correct point mutant (*Taz^PM^)* editing and a lack of residual targeting sequences in the genome were confirmed by Sanger sequencing and PCR to identify two positive founders. The *Taz^PM^* allele was detected using the following specific primers (forward-TGGCACAGTAAGAAGCCTCG and reverse-GGGACACACAAAACTTCCAGC), followed by restriction enzyme confirmation (as the *D75H* substitution introduces a new *AvaII* site, resulting in either positive digest 179 bp + 162 bp mutant bands in heterozygotes or a single uncut 341bp band in wild-types). As 100% of the surviving *Taz^PM^* hemizygous males (*Taz^PM^*♂) were infertile (*n* = 38/38), in contrast to unaffected *Taz^PM^* hemizygous females (*n* = 69/69), *Taz^wt^/Taz^PM^* females were crossed to wild-type males (*wt*♂) to generate affected *Taz^PM^/Y* male offspring. *Taz^PM^* mice were backcrossed with C57BL/6J mice (Jackson Labs) for >10 generations and housed individually to reduce stress and enable unfettered feeding, prior to analysis. Systemic knockout (*Taz^KO^*) mice [8] that lack *Taz* exons 5–10 were generated via crossing C57BL/6J male and female floxed *Taz^fl/fl^* with ubiquitously expressed *EIIa-Cre* mice and genotyped as previously described [8]. Sex-specific *Zfy* primers were used to confirm male genotypes (forward-GACTAGACATGTCTTAACATCTGTCC and reverse-CCTATTGCATGGACAGCAGCTTATG). Mice were weaned at 4–5 weeks of age, fed distilled water and standard rodent chow, and maintained under specific pathogen-free conditions with a 12-h light/dark cycle. The use of animals in this study conformed to the Guide for the Care and Use of Laboratory Animals (National Academies Press, 2011). Animal procedures and experimental conditions were refined to minimize harm to animals and performed with the approval of the Institutional Animal Care and Use Committee of Indiana University School of Medicine (approval reference #23095). 

### 4.3. Survival and Bodyweight

Kaplan–Meier survival plots for the *wt*♂ and *Taz^PM^*♂ mice were constructed from records that logged all mice survival endpoint dates that died natural deaths, and the survival rates were compared using the log-rank test. Body weight was measured biweekly after 14 days of age in each mouse and compared among the *wt*♂ and surviving *Taz^PM^*♂ mice.

### 4.4. Blood Tests

Blood was drawn from 4-week, 4-month, and 10-month *wt*♂ and *Taz^PM^*♂ littermate lateral tail veins and collected into EDTA-coated tubes. Complete blood counts were obtained using a Hemavet 950FS Hematology Analyzer (Drew Scientific, Plantation, FL, USA) and stained using Giemsa stain (Sigma, Burlington, VT, USA). Select samples were also spotted onto Whatman^®^ 903 protein saver cards for subsequent lipid analysis.

### 4.5. Mouse Embryonic Fibroblasts and Lentiviral Transfection

MEFs were harvested from C57BL/6J wild-type and *Taz*-deficient male E14 embryos and grown in DMEM high glucose complete media (Thermo Fisher Scientific, Waltham, MA, USA) with 2 mM glutamine and 10% fetal bovine serum, as described [70]. They were transfected with either wild-type *TAZ* or mutant *TAZ^D75H^* human cDNAs. To introduce the *D75H* mutation, *ΔExon 2* version of wild-type TAZ cDNA was amplified with primers (forward-GTCCTGCATGGACCACCCTCATCTCTG and reverse-TGGTGATTGGACACGGTGATGAG-3′) using High-Fidelity DNA Polymerase (New England Biolabs, Ipswich, MA, USA). To generate the transfer plasmid containing the human *TAZ* gene (pLJM1-hTAZ and –hTAZD75H), wild-type and *TAZ^D75H^* variant cDNAs were amplified using primers (forward-TATAGCTAGCATGCCTCTGCACGTGAAGTGG and reverse-TATTCGAACTATCTCCCAGGCTGGAGGTGG). PCR products were cloned into pLJM1-EGFP using primer-generated unique NheI/BstBI sites. Following harvesting of lentiviral-containing media, viral concentrations were determined by enzyme-linked immunosorbent assay with anti-p24 (QuickTiter^TM^ Lentivirus Titer Kit, Cell Biolabs, San Diego, CA, USA). Then 9 × 10^5^ of *TAZ*^KO^ MEFs (-/Y) were mixed with 10 mL of 293T Complete media containing 0.5–1 × 10^9^ particles/mL of human *TAZ* wild-type and *TAZ^D75H^* variant lentivirus and polybrene (MilliporeSigma, Burlington, VT, USA). After 60 h, the media was replaced with MEF, collected by trypsinization, and washed with PBS after an additional 48 h wash. Concentrated MEFs in PBS were spotted on Whatman 903 protein saver cards (MilliporeSigma). Duplicate aliquots were incubated at 70 °C and used for Western blot.

### 4.6. Liquid Chromatography Mass Spectrometry

Blood samples from 4-month *wt*♂ and *Taz^PM^*♂ littermates were spotted onto the Whatman protein saver card and samples were extracted via chloroform/methanol, vortexed, sonicated and samples evaporated in a 60 °C oven. Dried extracts were reconstituted with acetonitrile/isopropanol/water for analysis, as described [70]. For cardiolipin determination, lipid extracts were separated by reverse-phase HPLC in ZORBAX Eclipse Plus C18 2.1 × 50 mm, 1.8 μm particles (Agilent, Santa Clara, CA, USA) with solvent A (Acetonitrile/Water [60:40], 10 mM ammonium formate, 0.1% formic acid) and solvent B (Isopropanol/Acetonitrile [90:10], 10 mM ammonium formate, 0.1% formic acid) at 55 °C. Bound lipids were eluted, ionized, and analyzed via 6495A Triple Quadrupole LC/MS System (Agilent). Urine samples from 4-month and 12-month *wt*♂ and *Taz^PM^*♂ littermates were expressed directly into microcentrifuge tubes. Urine samples were frozen on dry ice and stored at −80 °C until thawed on an ice bath and filtered using 0.2 μm filters with a PTFE membrane (PALL Corporation, New York, NY, USA) prior to liquid chromatography–mass spectrometry (via the IU Mass Spectrometry Core). 3-methylglutaric acid and creatinine levels were analyzed using an Agilent Technologies 1100–6130A System with an Acquity UPLC BEH Amide column (100 mm × 2.1 mm, 1.7 μm; Waters Corporation, Milford, MA, USA).

### 4.7. Transthoracic Echocardiography

Left ventricular performance was examined by 2-dimensional echocardiography using a Vevo 2100 Ultrasound (Visual Sonics) in isoflurane-anesthetized mice (~30 s for induction at 4 vol% at 2-L oxygen flow). M-mode LV and 2D views were used to examine the structure and measure anterior and posterior wall thicknesses at the end diastole and end systole, as described [71]. The following parameters were also obtained: left ventricular internal diameter in either diastole (LVIDd) or systole (LVIDs), and cardiac output (CO). Fractional shortening (FS) was calculated according to the following formula (FS% = ([LVIDd − LVIDs]/LVIDd) × 100) and ejection fraction (EF) was calculated using (EF% = ([EDV − ESV]/EDV) × 100). A single operator blinded to mouse genotypes performed the echocardiography and data analysis.

### 4.8. Western Analysis

For Western blot analysis, transfected MEFs and microdissected *Taz^PM^*♂ and *wt*♂ ventricles (atria and valvular tissues removed) and individual organs (*n* = at least 4–8 of each genotype/stage/tissue/antibody) were homogenized in RIPA protein lysis buffer (Sigma) and resultant protein concentrations normalized as described [71]. Cytosolic and nuclear fractions were isolated according to Extraction Kit manufacturer instructions (Invent Biotechnologies, Plymoth, MA, USA). Mitochondrial fractions were isolated following Trypsin digestion and Sucrose extraction, as described [70]. Samples (~20–30 ug/lane) were resolved using SDS-PAGE (BioRad, Hercules, CA, USA) and transferred to PVDF Western Blotting Membranes. Following blocking in Blotting-Grade Blocker (Bio-Rad) blots were probed with the following antibodies: Acaa2 #237540, Arginase #96183, Bcl2 #16904, Bcl2-interacting protein-3 #109362, Catalase #209211, Cathepsin D #75852, CDKN2A/p16INK4a #211542, Connective tissue growth factor #209780, Galectin-3 #76245, Hadh #154088, Lactate dehydrogenase #52488, nuclear Lamin B1 #229025, Myoglobin #77232, Phospholamban #219626, Phospholamban (phospho S16) #15000, PKA alpha/beta/gamma (phospho T197) #75991, p21 #188224, p53 (phospho S15) #278683, p53 (acetyl K370) #183544, Serca2 #3625, Sequestosome-1 #109012, Sirt3 #246522, Sod2 #68155, TIGAR #62533, Ucp3 #193470; Slc38a1 (Bioss Antibodies #bs-19825R); MDM2 (BioRad #AHP1329); AMPK #2532, Caspase-3 #9662, cleaved Caspase-3 #9661, Cycs #4272, pCaMKII (phospho T286) #12716, Mcu #14997 (Cell Signaling); TP53INP2 (LSBio #LS-C413392); Atp5a1 (Proteintech #14676-1-AP); Acsl1 #PA5-17136, 4-HNE #MA5-27570, LC3B #PA1-46286, VDAC #PA1-954A (Invitrogen); Pla2g6 (Novus Biologicals #NBP1-81586); Ly-6G #sc-53515, p53 #sc-71820, Pten #sc-7974, Pink1 #sc-517353, Taz #sc-365810 (Santa Cruz), GAPDH #G8795, cytosolic Tubulin #T5168 (Sigma). Total OxPhos Rodent WB Antibody Cocktail (Abcam #ab110413) was used to analyze the relative levels of OxPhos complexes in *Taz^PM^*♂ and *wt*♂ mitochondrial LV fractions. Immunoreactive protein band signals were detected via ECL Western Blotting Detection (Amersham, Amersham, UK) with species-appropriate peroxidase-conjugated secondary antibodies (Bio-Rad and Jackson ImmunoResearch, West Grove, PA, USA). To verify equal loading, all blots were subsequently stripped (Fisher Stripping Buffer for 15 min at 37 °C), washed, re-blocked, and then probed with housekeeping GAPDH and/or Tubulin loading controls to monitor protein integrity. All X-ray films of varying exposures were scanned for each antibody, and densitometric signal intensity was quantified in *n* = 4–8 of each genotype/stage/tissue/antibody. Statistical analysis was performed via Prism software version 5.02 (GraphPad Software).

### 4.9. qPCR

Total RNA was isolated from triplicate 4-week and 6-month *wt*♂ and *Taz^PM^*♂ littermate ventricles (atria and valvular tissues removed) individually using Qiagen isolation columns following manufacturer’s recommendations. cDNA was synthesized in a 20 uL reaction with 1 ug of RNA using random primers (SuperScripTM III First-Strand Synthesis System for qPCR, Invitrogen). Three separate ventricle cDNAs were amplified in duplicate with candidate mouse-specific PCR primers and both *Gapdh* and *PPia* housekeeping genes (see Primer list in Table 3), as described [72]. Amplified PCR bands were cloned and sequenced to verify identity. Cycle number as a quantitative estimate of the initial template concentration in a sample, was quantified from at least *n* = 4 duplicate samples and normalized to both *Gapdh* and *PPia* housekeeping genes. Statistical analysis was performed via Prism software version 5.02 (GraphPad Software).

### 4.10. Histologic Analysis, Lineage Mapping, and Molecular Marker Analysis

Hearts and livers were harvested in ice-cold PBS and fixed with 4% paraformaldehyde (Sigma) prior to processing and embedding in Polyfin wax (General Data, Cincinnati, OH, USA) or OCT (Tissue-Tek, Torrance, CA, USA) for histological analysis, in situ hybridization or immunostaining. Serial sections (5–10 μm thickness) were stained with Hematoxylin/Eosin, Picro Sirius red/Methyl green stain, or Masson’s trichrome (Sigma-Aldrich, St. Louis, MO, USA) to assess histology and/or fibrosis. Frozen sections (5 μm thickness) were stained with Oil Red-O (Sigma-Aldrich), Wheat germ agglutinin, Alexa Fluro-488 conjugate to label cell membranes (Invitrogen, Waltham, MA, USA), senescence-associated β-galactosidase (Gold Biotechnology, Olivette, MO, USA) and Xgal as described [73] or used for immunohistochemistry and TUNEL labeling. For immunostaining, serial sections were first antigen retrieved via microwave in citrate pH6 buffer, then probed with the following primary antibodies: Cathepsin D, Galectin-3, Nppa, Postn, (Abcam, Cambridge, UK) LC3B (Invitrogen) and detected using the ABC kit and species-specific secondary antibodies (Vectorstain). Both DAB/hydrogen peroxide and fluorescence were used to visualize immunosignals. Antibody diluent (Vectorstain, Newark, NJ, USA), without primary antibodies, was used for negative controls. For each assay, serial sections were examined, and a specific signal was only noted when present in at least three consecutive serial sections. Cell apoptosis was evaluated using TdT-FragELTM DNA Fragmentation Kit (Calbochem, Burlington, VT, USA) following the manufactures protocol. For non-radioactive in situ hybridization, both sense and anti-sense non-radioactive *Bmp10* RNA probes (gift from W. Shou) were labeled with digoxigenin using the DIG RNA Labeling kit (Roche) as described [74]. Specific signal was only observed when sections were hybridized with the anti-sense probe, and serial sections were examined for comparable spatiotemporal patterns of *Bmp10* expression in at least three consecutive serial sections in at least three individual *wt*♂ and *Taz^PM^*♂ hearts. 

### 4.11. Transmission Electron Microscopy

For electron microscopy, ventricle samples were isolated and fixed in 2% paraformaldehyde, and 2% glutaraldehyde fixative in 0.1 M cacodylate buffer (pH 7.2) overnight, processed, and imaged on a Phillips400 microscope (via IU EM Core) as described [72]. Mitochondrial morphology and grid counting to estimate mitochondrial number per 0.1 mm^2^ field (15 fields/LV/genotype) were performed blinded to genotype and analyzed using ImageJ (version 1.54j). 

### 4.12. NAD^+^/NADH and ATP/ADP/AMP Measurement

Micro-dissected ventricles were immediately freeze-clamped in liquid nitrogen and stored at −80 °C until analysis. Frozen samples were homogenized in ice-cold 80% methanol/20% water using Kontes glass tissue grinders to remove protein. Ultra-performance liquid chromatography (UPLC) analyses were used to measure adenine nucleotides in the extracts with UV-Vis and mass spectrometry (MS) simultaneously, as described [75].

### 4.13. Statistical Analysis

Data are presented as mean ± SD from independent experiments with different biological samples per group. The following statistical tests were used: unpaired 2-tailed t-test for comparisons between 2 groups, 1-way ANOVA with Tukey-Kramer multiple-comparison test for multiple groups, Chi-squared analysis of contingency tables when the sample sizes are large, and the Fisher’s exact test when sample sizes are lower than 10. For the Unpaired two-tailed *t*-test, statistical significance was set at *p* < 0.05, *p* < 0.01, and *p* < 0.001.

## Figures and Tables

**Table 1:** Numbers of offspring of different genotypes at weaning.

Parental Cross: *wt* Male x *Taz^PM^* Female				
Genotype	Expected	Observed	Chi2 *p* Value	Fertile
*wt* ♂	70 (25%)	82 (29.4%)	0.252	100%
*Taz^PM^* ♂	70 (25%)	38 (13.6%)	**<0.0001**	0%
*wt* ♀	70 (25%)	67 (23.9%)	0.271	100%
*Taz^PM^* ♀	70 (25%)	69 (24.6%)	0.285	100%

**Table 2:** Physiological and echocardiographic indices in adult and juvenile offspring.

	Adult *Wt*♂ (*n* = 8)	Adult *Taz^PM^*♂ (*n* = 8)	Juvenile *Wt*♂ (*n* = 3)	Juvenile *Taz^PM^*♂ (*n* = 3)
Body weight (g)	39.6 ± 2.7	33.19 ± 3.4 *	26.7 ± 2.2	20.9 ± 1.3 *
Heart rate ^@^ (bpm)	487.3 ± 17.5	496.11 ± 37.7	467.9 ± 9.1	424.5 ± 29.9
LV mass (mg)	228.7 ± 22.1	189.1 ± 11.8 *	107.9 ± 17.6	424.5 ± 29.9
FS (%)	228.7 ± 22.1	18.9 ± 3.1 **	45.3 ± 2.5	35.6 ± 1.5 **
EF (%)	58.0 ± 1.4	38.7 ± 2.8 **	75.7 ± 2.8	65.0 ± 1.9 *
LVID_d_ (mm)	4.4 ± 0.1	3.2 ± 0.2 *	3.4 ± 0.1	3.7 ± 0.1
LVID_s_ (mm)	2.9 ± 0.4	1.7 ± 0.2 *	2.0 ± 0.1	2.5 ± 0.1
LV Vol_d_ (ml)	88.7 ± 3.2	128.7 ± 7.3 **	57.7 ± 3.8	61.9 ± 3.8
LV Vol_s_ (ml)	38.2 ± 4.2	59.0 ± 9.6 **	14.3 ± 2.3	22.4 ± 1.5 **

All values are mean ± S.E. Unpaired t-test, parametric significant differences between *Wt***♂** and *Taz^PM^*♂ groups are indicated. Abbreviations: Heart rate (beats/min (bpm)); LV, left ventricular mass; FS, left ventricular fractional shortening; EF, left ventricular ejection fraction; LVIDd, diastolic left ventricular internal dimension; LVIDs, systolic left ventricular internal dimension; LV Vold, left ventricular diastolic volume; LV Vols, left ventricular systolic volume. Notes: ^@^ were anesthetized heart rates, and statistical significance set at * *p* < 0.05 and ** *p* < 0.01 vs. *Wt***♂** mice.

**Table 3:** PCR primers used.

Gene	Forward PCR Primer	Reverse PCR Primer
*Gapdh*	AAGGGCATCTTGGGCTACAC	CATTGAGAGCAATGCCAGCC
*PPia*	GGGTGGTGACTTTACACGCC	CTTGCCATCCAGCCATTCAG
*Taz*	TGTGTCGAGGAGATGGTGTC	ACACTCAGCAATCAGCCGTC
*Prkaa1*	CCAAGGGGCATCTTGGGAAA	TCCTCCCAACAACGGCTTAC
*Prkaa2*	CACGGTGTGGTCGTTAGTGA	AGCATGTGGTACAAGCCCAG
*Lc3b*	TTGTCATCGTGGGAACTGGG	GGTGGCAAGGTATCGACCAA
*p62*	GGCCTACAGCTCCTAGGGAA	GAAGCTACATGGGGGTCCTG
*Bnip3*	TACCCACGAACCCCACTTTG	GCCTGCAACAAAACTGACCA
*p21*	TACCGTGGGTGTCAAAGCAC	GAGGACTCGGGACAATGCAG
*p16*	GACCGACGGGCATAGCTTC	AAGAAAAAGGCGGGCTGAGG
*p53*	CTCAGACTGACTGCCTCTGC	ACTACTCAGAGAGGGGGCTG
*Mdm2*	GGCATGAATTGCATCTGGTGG	ACCTGGGACAAACTGCAACA
*Tigar*	TGAGGAAATGCAGGGTGAGC	AACACTGCTGCTCAGATGCT
*Tp53inp2*	ATGAAGTGGATGGCTGGCTC	CTGCCGGTGACATAAACGGA

## Data Availability

Values for all individual data points are reported in the Supplement figures. All relevant data are available from the corresponding author upon request.

## Supplementary Materials

The following supporting information can be downloaded at: https://www.mdpi.com/article/10.3390/ijms25158201/s1.
