# Supplementary material for: A Barth Syndrome Patient-Derived D75H Point Mutation in TAFAZZIN Drives Progressive Cardiomyopathy in Mice
Source: Int J Mol Sci. 2024 Jul 27;25(15):8201. doi: 10.3390/ijms25158201 (PMC11311365; doi:10.3390/ijms25158201)
Supplement: Supplementary file 1 [file ijms-25-08201-s001.zip › ijms-3105639-supplementary.pdf]

Supplemental Materials & Methods

A Barth Syndrome Patient-Derived *D75H* Point Mutation in *TAFAZZIN* Drives Progressive Cardiomyopathy in Mice

Paige L. Snider [1,†], Elizabeth A. Sierra Potchanant [1,†], Zejin Sun [1], Donna M. Edwards [1], Ka-Kui Chan [1], Catalina Matias [2], Junya Awata [3], Aditya Sheth [1], P. Melanie Pride [1], R. Mark Payne [1], Michael Rubart [1], Jeffrey J. Brault [2], Michael T. Chin [3], Grzegorz Nalepa [1,‡] and Simon J. Conway [1,*]

1 Herman B. Wells Center for Pediatric Research, Indiana University School of Medicine, Indianapolis, IN 46033, USA; psnider@iu.edu (P.L.S.); esierra@iu.edu (E.A.S.P.); sunzejin1@yahoo.com (Z.S.); dmed@med.umich.edu (D.M.E.); kakuchan@iu.edu (K.-K.C.); adisheth@iu.edu (A.S.); ppride@iu.edu (P.M.P.); rpayne@iu.edu (R.M.P.); mrubartv@iu.edu (M.R.); gnalepa@iu.edu (G.N.)

2 Department of Anatomy, Cell Biology and Physiology, Indiana University School of Medicine, Indianapolis, IN 46202, USA; cmatias@iu.edu (C.M.); jebrault@iu.edu (J.J.B.)

3 Molecular Cardiology Research Institute, Tufts Medical Center, Boston, MA 02111, USA; j_awata@charter.net (J.A.); michael.t.chin@tuftsmedicine.org (M.T.C.)

* Correspondence: siconway@iu.edu; Tel.: +(317)-278-8780

† These authors contributed equally to this work.

‡ Deceased.

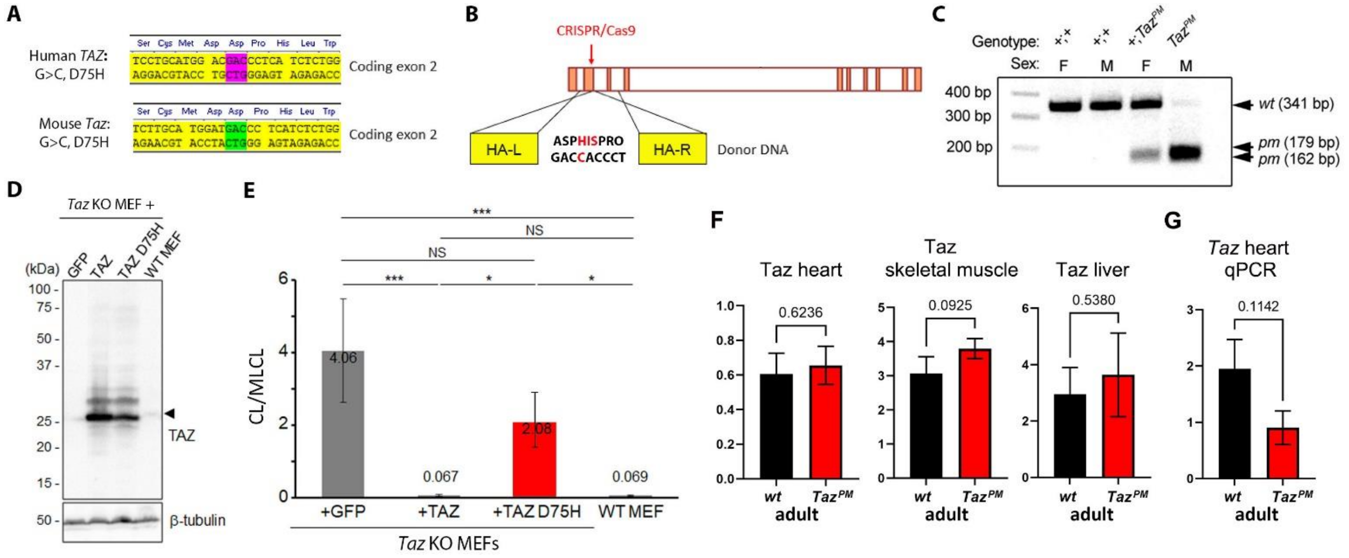

Supplemental Figure S1. Generation of *Taz^{PM}*♂ point mutant knockin mice. A, Conservation at *TAZ^{D75H}* mutation site in human and mouse *TAZ* exon 2. **B,** Schematic of *D75H* point mutation insertion strategy via *CRISPR/Cas9* targeting. Both the left (HA-L) and right (HA-R) homology arms of the donor DNA are indicated, along with the *CTP* point mutation. **C,** Representative Polymerase chain reaction (PCR) genotyping of male (M) and female (F) *wt* and *Taz^{PM}* offspring, following *AvaII* restriction enzyme confirmation. Note the absence of a *wt* 341bp band in only *Taz^{PM}*♂ DNA. **D,** Western blot of whole cell extracts of *Taz^{PM}*♂ null MEFs transduced with lentiviruses expressing *GFP* only, human wildtype *TAZ,* or human point mutant *TAZ^{D75H}* cDNAs versus whole cell lysates from *wt*⚦ MEFs. Lysates are probed with validated anti-Taz antibody (Santa Cruz F-7) that detects both human and mouse Tafazzin. Note the expression levels of both wildtype TAZ and TAZ^{D75H} exogenous proteins are comparable and much higher than endogenous murine Taz (arrowhead). **E,** Quantification of MLCL/CL ratio in cultured *wt*♂ and *Taz*♂ null MEFs transduced with *GFP*, human *TAZ* and *TAZ^{D75H}* lentiviruses. Significantly, the elevated MLCL/CL ratio caused by absence of TAZ is fully rescued by normal TAZ, but not by TAZ^{D75H} mutant (red) or GFP transfected proteins. Values were determined in replicate experiments (n=4) and means ±SD statistical significance determined by the Tukey-Kramer method after one-way ANOVA: NS, $p \geq 0.05$; * $p<0.05$; *** $p<0.001$. **F,** Quantitative and statistical analysis of Taz protein levels in *wt* and *Taz^{PM}*♂. Bands were densitometrically quantified and relative expression normalized to GAPDH control levels (n=5 of each genotype/organ). **G**, *Taz* PCR cycle amplification numbers were normalized to *Gapdh* and *PPia* housekeeping control levels to generate a quantitative estimate of the initial template concentration in each sample (n=4/genotype). Analysis of Western and qPCR results were carried out using 2-tailed Student's t tests and statistical significance was set at * $P<0.05$.

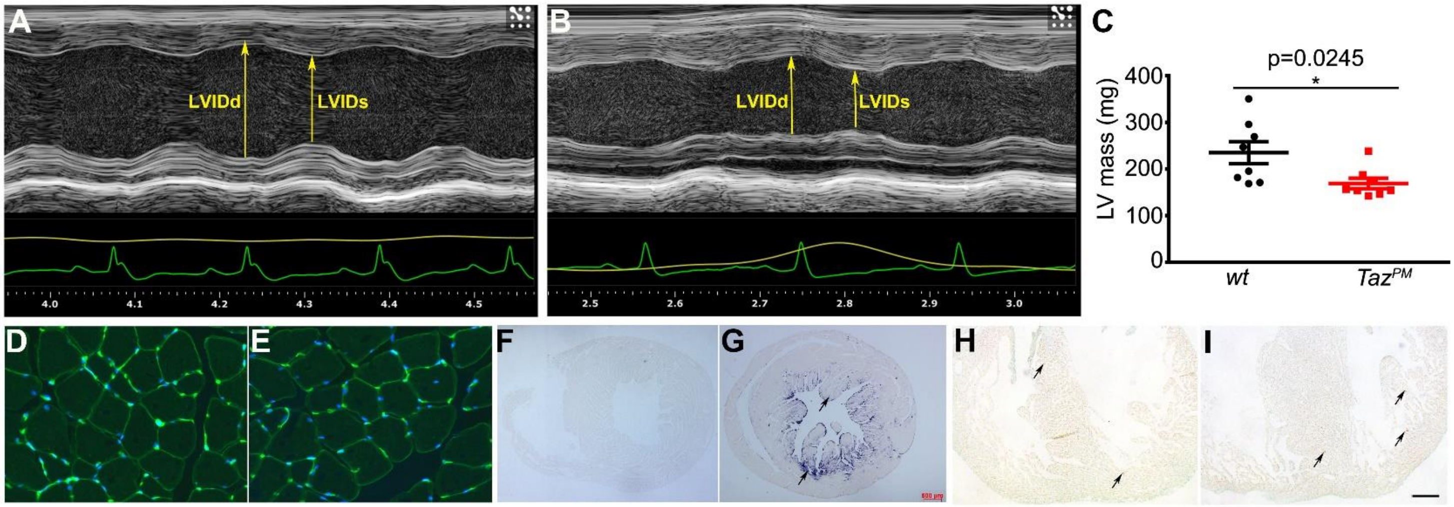

Supplemental Figure S2. Phenotyping *Taz*^{PM}♂ hearts. **A,B,** Echocardiographic images of LV short-axis M-mode assessment of 10 month *wt*♂ (**A**) and *Taz*^{PM}♂ (**B**) cardiac function (n=8/genotype). Note both LV internal diameter during systole (LVIDs) and diastole (LVIDds) were reduced in *Taz*^{PM}♂ vs *wt*♂ adults. LVIDd is 4.42+/-0.07mm in *wt*♂ vs. 3.18+/-0.22mm in *Taz*^{PM}♂; LVIDs is 2.89+/-0.04mm in *wt*♂ vs. 1.73+/-0.26mm in *Taz*^{PM}♂ (p=0.05). **C,** LV mass is decreased ~40% in *Taz*^{PM}♂ compared to *wt*♂ littermates (n=8/genotype, p=0.024). **D,E,** Wheat germ agglutinin staining of adult *wt*♂ (**D**) and *Taz*^{PM}♂ (E) cross-sectional cardiomyocyte sizes (n=5 genotype/age), x400 mag. **F,G,** Non-radioactive *in situ* hybridization revealed ectopic *Nppa* mRNA is induced in mainly the adult *Taz*^{PM}♂ trabecular LV zone (G, arrow). **H,I,** Sparse TUNEL staining (arrows) is comparable in 4-week juvenile *wt*♂ (F) and *Taz*^{PM}♂ (G) heart serial sections (n=8 genotype/age). Similarly, there is no elevation in TUNEL-positive staining in adult nor neonatal *Taz*^{PM}♂ hearts (n=5 genotype/age; negative data not shown). Scale bars: F,G=500μm, H,I=100μm.

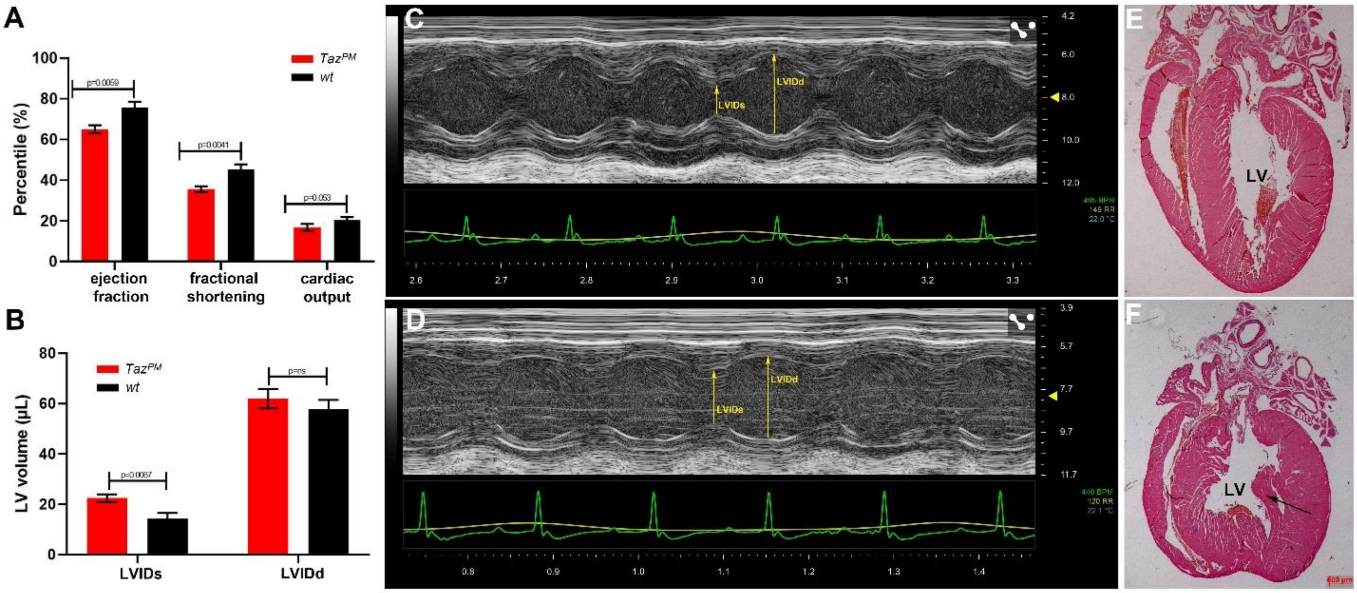

Supplemental Figure S3. Echocardiographic and histological analysis of juvenile *Taz*^{PM} male hearts. A, Quantification of M-mode LV function revealed that both ejection fraction (EF, p=0,006) and fractional shortening (FS, p=004) were significantly decreased in *Taz*^{PM}♂ (red) compared to *wt*♂ (black) juvenile littermates. Further, reduced cardiac output (CO, p=0.063) in *Taz*^{PM}♂ is trending significant compared to *wt*♂ (n=3/genotype). **B,** Further, analysis of LV volumes indicates there is LV dilation in *Taz*^{PM}♂ systole but not diastole, as LVIDs is enlarged but LVIDd is unchanged in *Taz*^{PM}♂ vs *wt*♂ juveniles. **C,D,** Representative echocardiographic images of LV short-axis M-mode assessment of juvenile *wt*♂ (C) and *Taz*^{PM}♂ (D) cardiac function (n=3/genotype). **E,F,** Histology confirmed all (n=3/3) juvenile *Taz*^{PM}♂ (F) exhibit a dilated “pumpkin shaped” heart with LVNC, compared to *wt*♂ (E) littermates. Prominent LV trabeculations (F, arrow) are only present in *Taz*^{PM}♂ hearts. Abbreviation: LV, left ventricle. Scale: E,F=500μm.

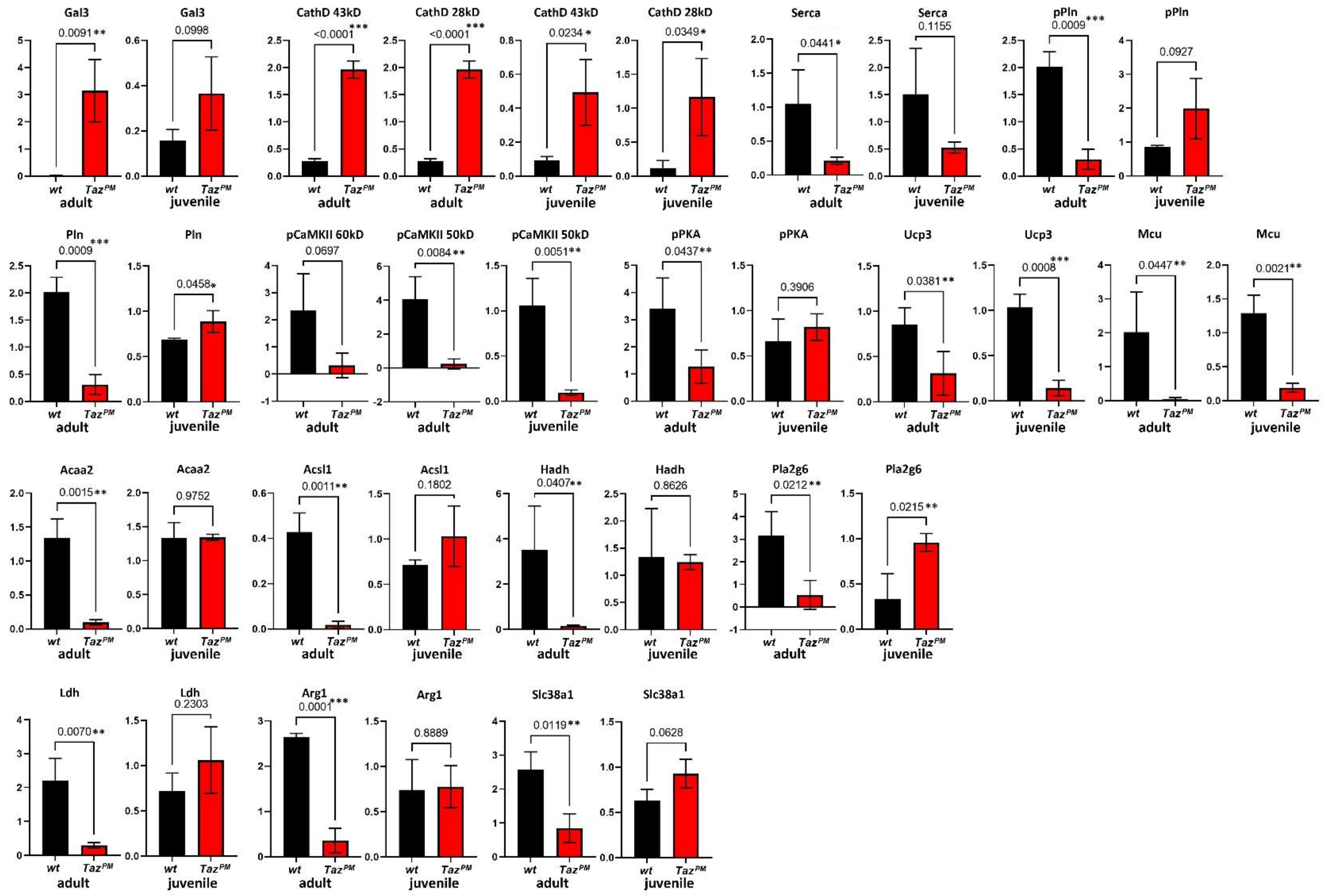

Supplemental Figure S4. Quantitative and statistical analysis of progressive cardiomyopathy protein biomarkers in *wildtype* and *Taz*^{PM} male ventricles. Bands were densitometrically quantified and relative expression normalized to GAPDH housekeeping control levels. Analysis was performed on n=4-8 of each genotype/stage/antibody, with statistical analysis carried out using 2-tailed Student's t tests. Statistical significance was set at * P<0.05, ** P<0.01 and *** P<0.005.

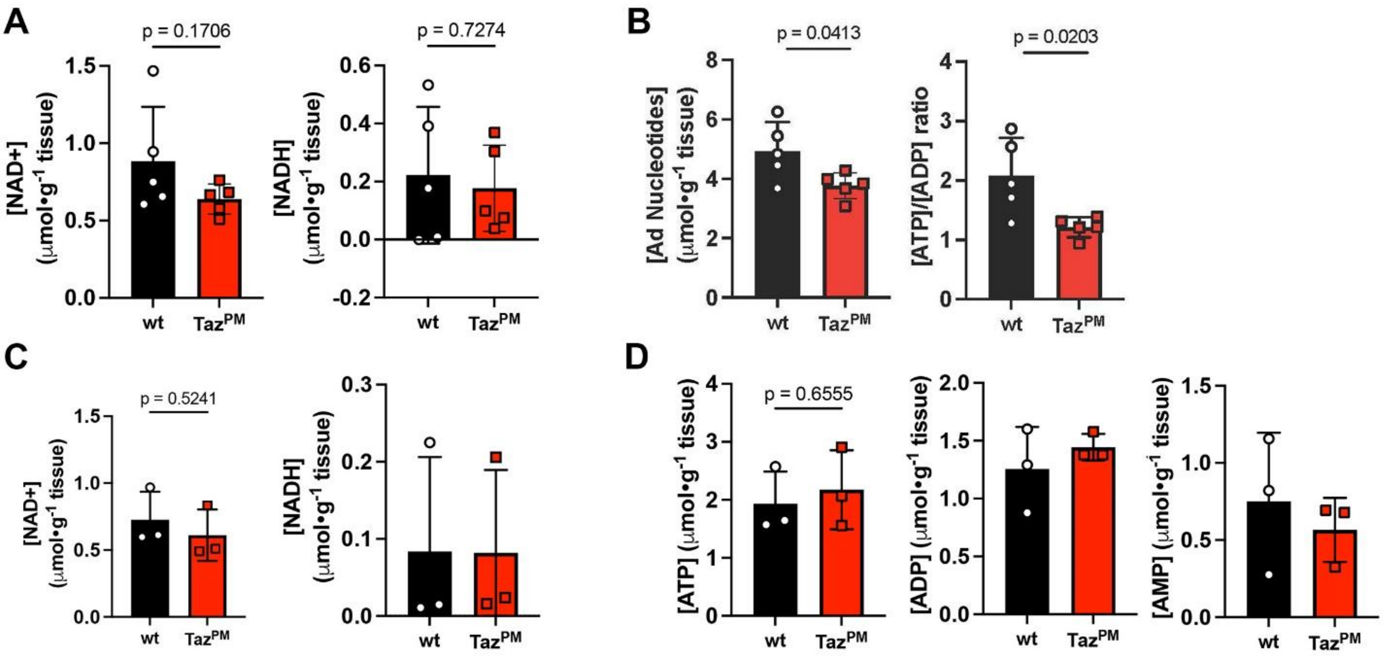

Supplemental Figure S5. Adenine nucleotide and nicotinamide adenine dinucleotide Ultra-performance liquid chromatography analysis. A, Surviving adult *Taz*^{PM}♂ NAD$^+$ levels trend ~14% lower (p=0.17), however NADH levels were not significantly unaffected (n=5/genotype). **B,** Surviving adult *Taz*^{PM}♂ total adenine nucleotide pool is reduced (p=0.0413) compared to *wt*♂ littermates and the ATP/ADP ratio is also significantly reduced (p=0.02) in *Taz*^{PM}♂ (n=5/genotype). **C,D,** Surviving juvenile *Taz*^{PM}♂ NAD$^+$ and NAHD levels (C) and ATP, ADP and AMP levels (D) were unaltered when compared to *wt*♂ littermates (n=3/genotype).

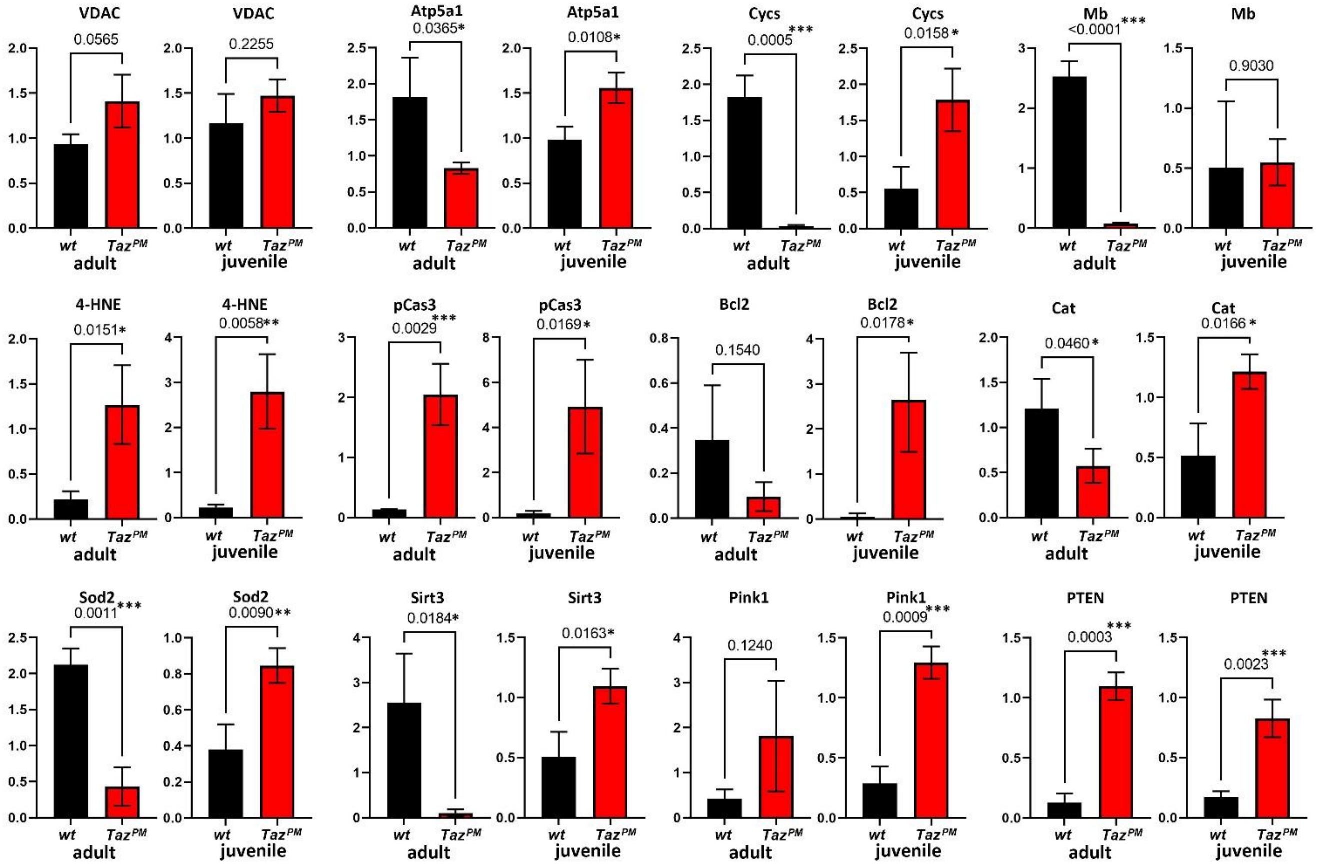

Supplemental Figure S6. Quantitative and statistical analysis of mitochondrial effectors in *wildtype* and *Taz*^{PM} male ventricles. Bands were densitometrically quantified and relative expression normalized to GAPDH housekeeping control levels. Analysis was performed on n=4-8 of each genotype/stage/antibody, with statistical analysis carried out using 2-tailed Student's t tests. Statistical significance set at * P<0.05, ** P<0.01 and *** P<0.005.

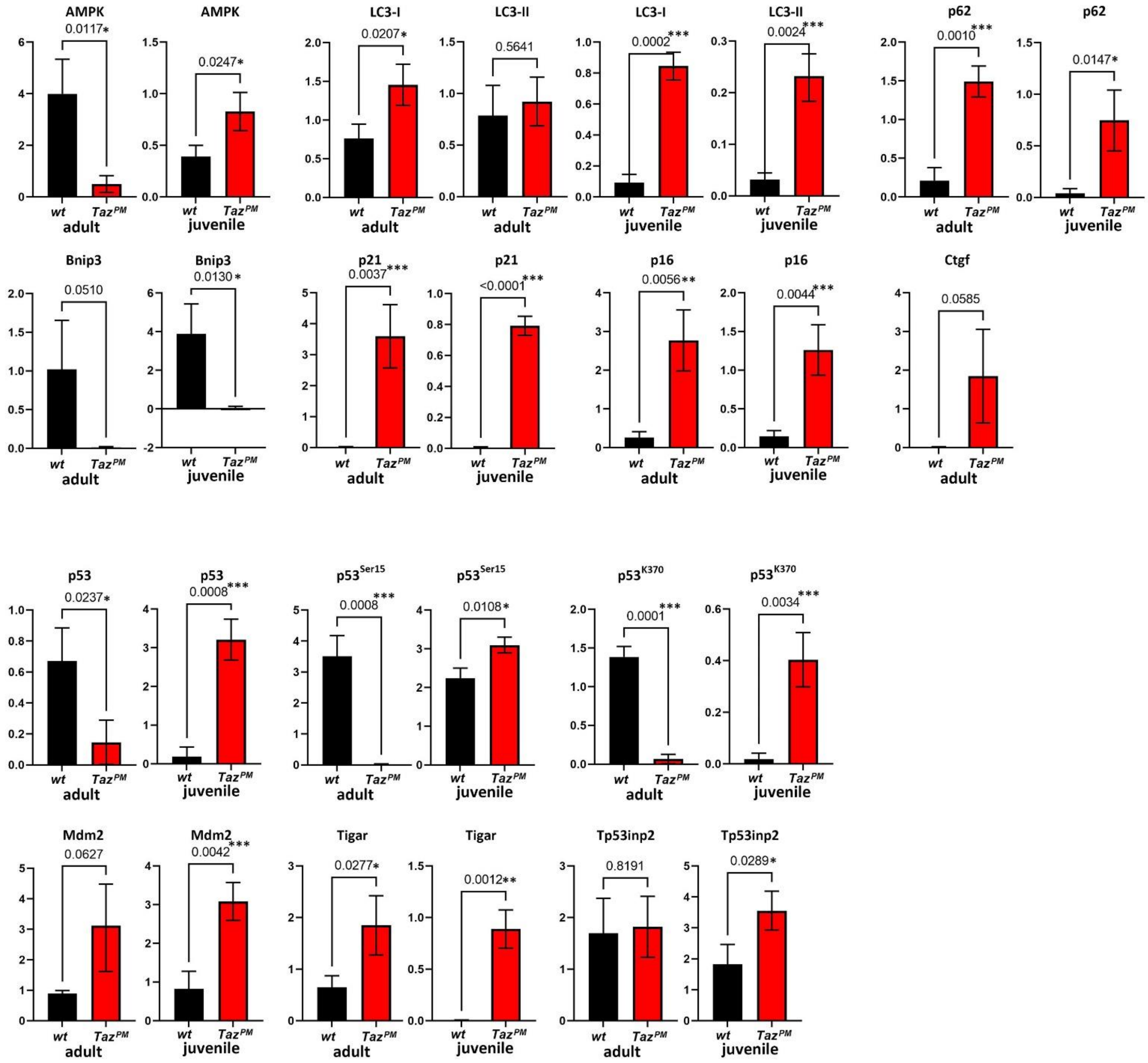

Supplemental Figure S7. Quantitative and statistical analysis of autophagy-mediated senescence and p53 pathway biomarkers in *wildtype* and *Taz*^{PM} male ventricles. Bands were densitometrically quantified and relative expression normalized to GAPDH housekeeping control levels. Analysis was performed on n=4-8 of each genotype/stage/antibody, with statistical analysis carried out using 2-tailed Student's t tests. Statistical significance set at * P<0.05, ** P<0.01 and *** P<0.005.

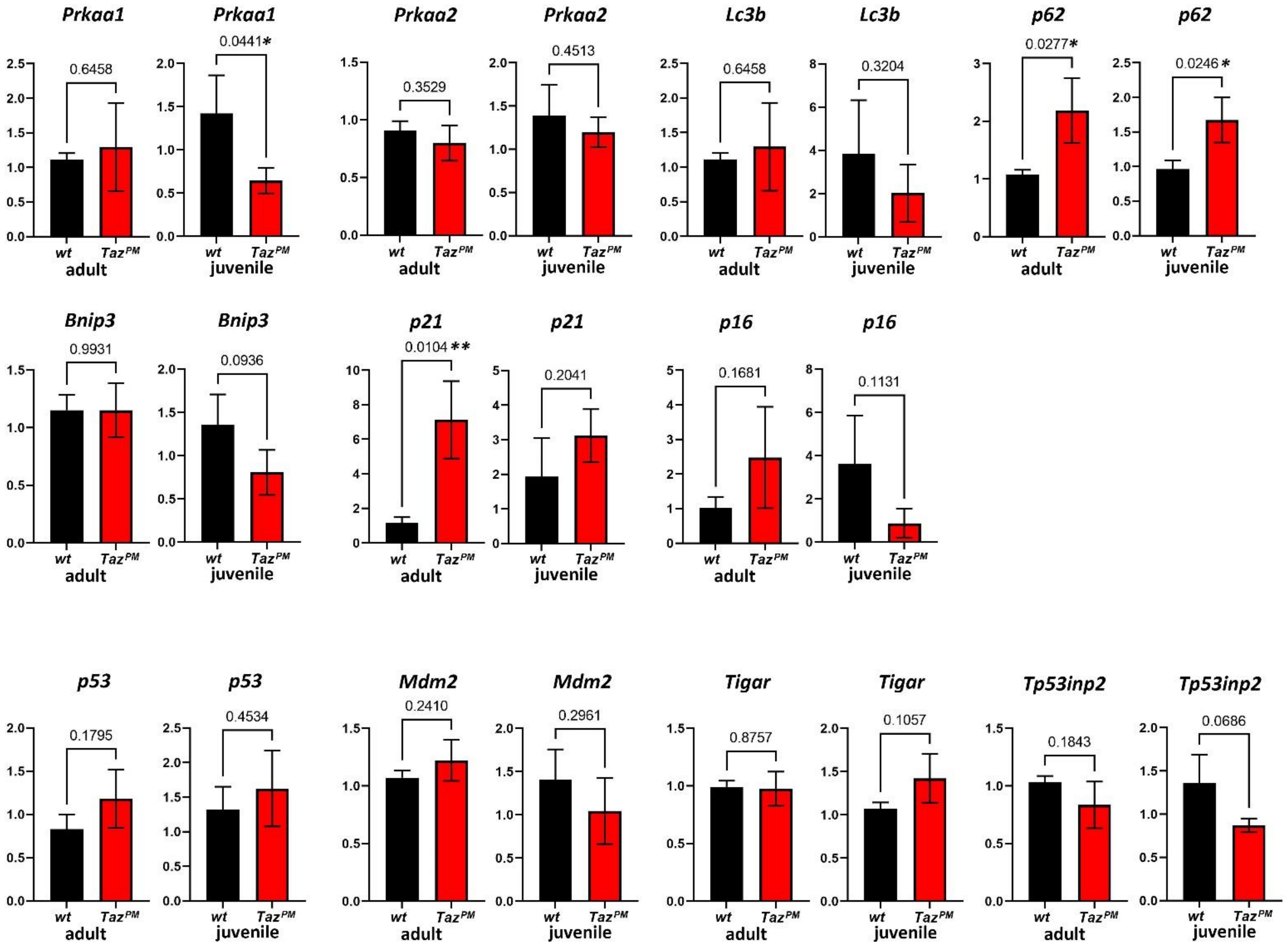

Supplemental Figure S8. Quantitative analysis of autophagy-mediated senescence and *p53* pathway mRNA expression levels in *wildtype* and *Taz*^{PM} male ventricles. PCR cycle amplification numbers were normalized to *Gapdh* and *PPia* housekeeping control levels to generate a quantitative estimate of the initial template concentration in each sample. Analysis was performed on n=4 of each genotype/stage/gene, with statistical analysis carried out using 2-tailed Student's t tests. Statistical significance set at * P<0.05 and ** P<0.01.

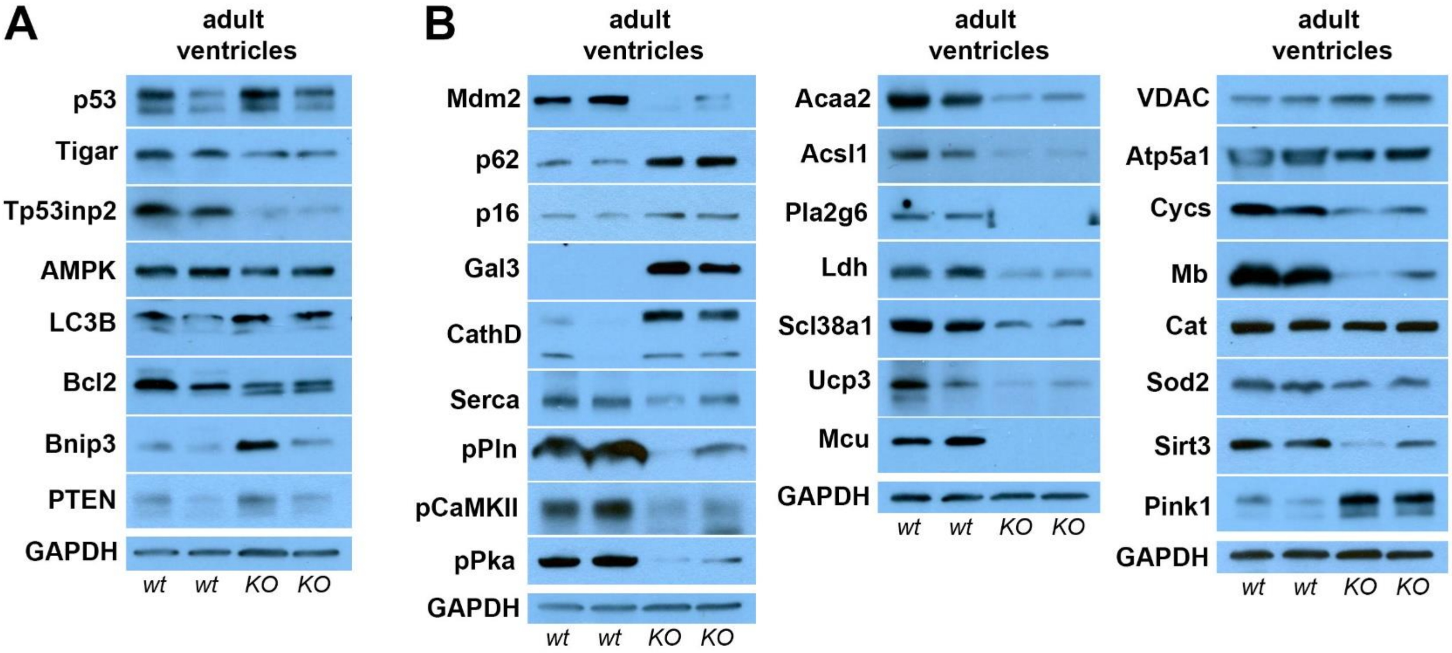

Supplemental Figure S9. p53 pathway is not dysregulated in *Taz*^{KO} null hearts. A, Representative western blots showing p53 and key p53 effector expression levels within duplicate *wt*♂ and *Taz*^{KO}♂ adult hearts that diverge from adult *Taz*^{PM}♂ (n=3/genotype/age). GAPDH was used as a loading control. **B,** Representative western blots showing equivalent adult *Taz*^{PM}♂ altered expression levels of key heart failure, metabolism and mitochondrial effectors in duplicate *wt*♂ and *Taz*^{KO}♂ adult hearts (n=3/genotype/age). GAPDH was used as a loading control.
